# Estimated EEG functional connectivity and aperiodic component induced by vagal nerve stimulation in patients with drug-resistant epilepsy

**DOI:** 10.3389/fneur.2022.1030118

**Published:** 2022-11-23

**Authors:** Roberta Coa, Simone Maurizio La Cava, Giulia Baldazzi, Lorenzo Polizzi, Giovanni Pinna, Carlo Conti, Giovanni Defazio, Danilo Pani, Monica Puligheddu

**Affiliations:** ^1^Neuroscience Ph.D. Program, Department of Biomedical Sciences, University of Cagliari, Cagliari, Italy; ^2^Department of Electrical and Electronic Engineering, University of Cagliari, Cagliari, Italy; ^3^Department of Informatics, Bioengineering, Robotics and Systems Engineering, University of Genova, Genova, Italy; ^4^Regional Center for the Diagnosis and Treatment of Adult Epilepsy, Neurology Unit, AOU Cagliari, Cagliari, Italy; ^5^SC Neurosurgery, Neuroscience and Rehabilitation Department, San Michele Hospital, ARNAS G. Brotzu, Cagliari, Italy; ^6^Department of Medical Sciences and Public Health, University of Cagliari, Cagliari, Italy

**Keywords:** vagus nerve stimulation (VNS), EEG, functional connectivity (Fc), aperiodic component, drug-resistant epilepsy (DRE)

## Abstract

**Background:**

Vagal nerve stimulation (VNS) improves seizure frequency and quality of life in patients with drug-resistant epilepsy (DRE), although the exact mechanism is not fully understood. Previous studies have evaluated the effect of VNS on functional connectivity using the phase lag index (PLI), but none has analyzed its effect on EEG aperiodic parameters (offset and exponent), which are highly conserved and related to physiological functions.

**Objective:**

This study aimed to evaluate the effect of VNS on PLI and aperiodic parameters and infer whether these changes correlate with clinical responses in subjects with DRE.

**Materials and methods:**

PLI, exponent, and offset were derived for each epoch (and each frequency band for PLI), on scalp-derived 64-channel EEG traces of 10 subjects with DRE, recorded before and 1 year after VNS. PLI, exponent, and offset were compared before and after VNS for each patient on a global basis, individual scalp regions, and channels and separately in responders and non-responders. A correlation analysis was performed between global changes in PLI and aperiodic parameters and clinical response.

**Results:**

PLI (global and regional) decreased after VNS for gamma and delta bands and increased for an alpha band in responders, but it was not modified in non-responders. Aperiodic parameters after VNS showed an opposite trend in responders vs. non-responders: both were reduced in responders after VNS, but they were increased in non-responders. Changes in aperiodic parameters correlated with the clinical response.

**Conclusion:**

This study explored the action of VNS therapy from a new perspective and identified EEG aperiodic parameters as a new and promising method to analyze the efficacy of neuromodulation.

## Introduction

Vagal nerve stimulation (VNS) is a non-medical treatment for subjects with drug-resistant epilepsy (DRE), in whom surgical treatment is not feasible because of the multifocal, bilateral, or generalized origin of epilepsy or because of subject refusal or when surgery has had no curative effect with recurrence of seizures after surgery (1).

Although its safeness (2) and clinical efficacy on seizure frequency (3) and quality of life (4) in drug-resistant subjects have been demonstrated, the exact mechanism of action of VNS remains unclarified and is under constant exploration. An association between the induction of neuronal plasticity and the antiepileptic effect has been hypothesized but has not been fully demonstrated in humans (5–7).

The application of graph theory and network science to the characterization of the human brain (8) led to hypothesizing the existence of an intrinsic organization aimed at ensuring its efficiency (9). Although with limitations represented by volume conduction and the effects of field diffusion compared with source studies (10), surface neurophysiological techniques support the study of functional connectivity (Fc) between different neuronal groups underlying the electroencephalogram (EEG) electrodes placed on the scalp. Fc describes statistical dependency patterns of different electrodes in a selected time series (11) and is highly time-dependent because it is modulated by external stimuli (12).

The analysis of Fc can be performed on imaging examinations [i.e., functional magnetic resonance imaging (fMRI)] or neurophysiological studies [i.e., EEG and magnetoencephalography (MEG)] (9). The results obtained with these methods are different in many aspects and are not comparable because fMRI has a good spatial resolution (millimeters) but a poor temporal resolution (seconds), and EEG and MEG have a greater temporal resolution at the cost of a limited spatial resolution (8, 9). The use of a large number of electrodes, such as in 64-channel EEG systems, allows the increase in the EEG spatial resolution while maintaining an excellent temporal resolution.

Several Fc metrics have been proposed to study the impact of therapies on EEG signals (13); among these metrics, the phase lag index (PLI) quantifies the asymmetry of the distribution of phase differences between the two-time series and represents the synchronization levels of the EEG signal (14). Moreover, PLI is affected by possible interferences on the scalp-derived signal to a limited extent (15). Previous studies by our group, which focused on 21-channel EEG, have shown the action of VNS on Fc, specifically PLI (16–18).

However, the analysis of EEG based on classic frequency bands (i.e., alpha, beta, gamma, delta, and theta) does not represent the entire electrical activity of the brain (19); recent studies have shown the existence of underlying aperiodic components of the EEG signal that is related to biological functions, such as aging (20), cognitive fluctuations (21), and processes of neuronal excitation and inhibition (22), and diseases, for example, attention deficit hyperactivity disorder (23), schizophrenia (24), and sleep epilepsy (25). The aperiodic component, characterized by a 1/f-like power spectrum, can be characterized by parameters, including the exponent (representing the slope of the exponential reduction in power across frequencies) and offset (representing the shift in power across frequencies) (26). These parameters are highly conserved in individuals under different experimental conditions (27).

Although the effects of VNS on Fc parameters had already been observed (16–18, 28–31), no study has focused on the action of VNS on aperiodic components of the EEG.

Considering the previous studies on PLI (16–18), in this work, based on the hypothesis that VNS acts on synaptic plasticity, we aimed to evaluate the effect of chronic VNS therapy on the Fc estimated from scalp-recorded 64-channel EEG signals and its effect on aperiodic parameters to assess the potential correlation between these changes and the clinical outcomes of subjects with DRE. Specifically, we aimed to investigate the influence of VNS therapy on brain connectivity and EEG background activity both in responders and non-responders.

## Materials and methods

The Research Was Conducted in Accordance With the Declaration of Helsinki and Good Clinical Practice According to the International Conference on Harmonization Guidelines. The Study Was Approved by the Ethics Committee of the AOU Cagliari Which Reviewed the Trial Protocol, Amendments, and Patient-Informed Consent Form (PROT. PG/2019/6256). Written Consent Was Obtained From Each Patient Before Participation in the Study.

### Patient selection

For the aims of the study, candidates for VNS treatment due to DRE have been selected from the Regional Center for the Diagnosis and Treatment of Epilepsy at the University Hospital Duilio Casula of Monserrato (Italy).

The inclusion criteria were the diagnosis of DRE [according to the ILAE definition (32)] and the non-feasibility of surgical treatment. The exclusion criteria were psychiatric comorbidity and reduced compliance with the 64-channel EEG recording.

Clinical response to VNS was assessed by Labar's index (33) and McHugh's classification (34).

### EEG recording, epoch extraction, and processing

Each subject underwent a 40-min EEG recording in the morning while seated in a quiet room, in awakening and resting state, and during a seizure-free period, both clinical and electrical (at least 24 h away from the last generalized seizure). The whole examination lasted for 90 min, including the montage. During the recording, the operator constantly checked the subject's vigilance status. Recordings were performed before VNS implantation and 1 year after with ongoing stimulation. No changes in antiepileptic drug therapy were made during this period.

EEG signals were acquired by using a 64-channel EEG system (Micromed, Italy), with 61 channels placed on the scalp in accordance with the International 10–10 system for EEG signal, one channel for ECG (lead I), and two channels for electromyographic (EMG) polygraphy (belly-tendon montage on chin and neck muscles). EEG datasets were sampled at 1,024 Hz and bandpass filtered between 0.5 and 70 Hz.

EEG signals were saved in European Data Format (EDF) files and then visually analyzed by using an *ad-hoc* graphical interface developed in MATLAB (The Mathworks, MA, USA) that allowed data import, epoch selection, and signal processing.

In consideration of the Fc measure used (35), 8 s epochs (8,192 samples) were selected. Specifically, on each trace, 20 epochs free of artifacts and interictal abnormalities were selected by a neurologist experienced in clinical electroencephalography by visual inspection (R.C. and M.P.). To avoid possible interference of EMG activity on high EEG frequencies (36), we applied special care in the selection of epochs free of muscle artifacts. The EEG signal was first re-referenced to the common average, including all 61 EEG channels. Then, bandpass filters were applied to extract the five standard frequency bands (i.e., delta, theta, alpha, beta, and gamma). The digital implementation of the bandpass filters used in this study is detailed hereafter. All filters were implemented in the frequency domain. Specifically, for the delta, theta, alpha, beta, and gamma rhythms, we considered the 0.5–3, 3–8, 8–12, 12–30, and 30–50 Hz bands, respectively. Transition bands around the low and high cut-off frequencies of each bandpass filter were selected to be equal to 1 and 2 Hz, respectively, for the delta rhythm, 2 and 3 Hz for the theta rhythm, 3 and 4 Hz for the alpha rhythm, 4 and 5 Hz for the beta rhythm, and 3 Hz for both sides in the case of the gamma rhythm. After filtering, the selected epochs were exported in.txt format and saved for further analysis. All the subsequent analyses were performed by means of Athena, a publicly available toolbox developed in MATLAB (The Mathworks, MA, USA); the toolbox allows the extraction and analysis of measures commonly used for studying neural time series, such as EEG and MEG (https://github.com/smlacava/Athena).

### PLI

PLI is an Fc measure of the asymmetry related to the distributions of the phase differences between two signals, and it reflects the coupling between these time series, searching for a constant phase delay in the asymmetry of instantaneous phase differences (14).

For each subject, frequency band, and epoch, PLI was extracted from the EEG time series. From the obtained features, we computed the global values by averaging those related to single pairs of channels and within single epochs for each subject and each frequency band. The same procedure was repeated to obtain the values representative of single scalp regions (frontal, temporal, central, parietal, and occipital), averaging the values related only to the pairs of channels within each region. Finally, the value corresponding to each channel was computed as the average of all connectivity values related to the pairs of channels, including those of interest.

### Aperiodic parameters

Aperiodic parameters characterize the aperiodic activity as a 1/*f*^χ^ function, where χ is the exponent parameter, reflecting the exponentially decreasing pattern of aperiodic power across frequencies, and the offset parameter reflects the y-intercept in the log-transformation of such function, hence representing the uniform shift of power across frequencies (26).

For each subject and epoch, the two aperiodic parameters were extracted from the EEG time series.

For both parameters, we computed the global values by averaging those related to single channels and within single epochs for each subject. The same procedure was repeated to obtain the values representative of the single scalp regions (frontal, temporal, central, parietal, and occipital), averaging the values related only to the channels within a given region.

### Statistical analysis

To investigate the effects of VNS on responders and non-responders, we performed a statistical analysis on single features, that is, either the Fc or aperiodic parameters, through the Mann–Whitney *U*-test. We applied the false-discovery rate (FDR) correction (37) to the results, such that each of the corrected *p*-values was considered to be related to a significant test if it was lower than an α-value equal to 0.05.

We analyzed the PLI and aperiodic parameters separately, evaluating each of them for the effect of VNS on a single group of subjects (i.e., responders and non-responders) by comparing pre- and post-VNS implantation values of these measures.

Hence, we analyzed the PLI global values, representing the average values related to all pairs of channels and the values in regions, representing the average values related to all the pairs of channels within single regions of the scalp and the values in single channels, and representing the average values related to all pairs of channels including the considered channel. FDR correction was applied by considering all the frequency bands within the single spatial subdivision in each statistical analysis on Fc.

Likewise, we analyzed the exponent and offset aperiodic parameters for responders and non-responders separately, in terms of their global values, representing the average of values in the whole scalp, their averaged values in single regions, and their single-channel value. In all the statistical analyses on aperiodic parameters, the FDR correction was applied within the single spatial subdivision.

Finally, we analyzed the Spearman's rank correlation coefficient between the Labar's index related to each subject (which is the opposite of the percentage variation in seizure frequency) and the variation in the considered parameter (corrected through FDR) on the same subject to investigate the possible relationship between clinical response and the variation of these parameters after VNS implantation. Specifically, each variation was calculated for each subject as the difference between the median of values relative to the EEG recording after and those before VNS implantation divided by the latter to obtain relative variations.

## Results

Among the 18 selected subjects with DRE, 13 fit the inclusion and exclusion criteria and underwent EEG recording before implantation. VNS therapy was titrated as per technical data sheet indications over 6–8 weeks after implantation until an output current between 1.50 and 2 mA was reached, depending on the tolerability and clinical response of the individual patient; stimulation parameters for individual patients are shown in Table 1. However, three patients could not undergo EEG recording after 1 year due to the COVID-19 pandemic. The remaining 10 subjects studied comprised six men and four women aged between 27 and 61 years (average age: 41.9 years), of which seven had focal epilepsy and three had Lennox–Gastaut syndrome (LGS). The subjects were divided into responders (reduction of at least 50% of seizures after VNS, McHugh Classes I and II, *n* = 5) and non-responders (reduction of <50% of seizures, McHugh Classes III-V, *n* = 5) (Table 1).

**Table 1:** Clinical and registry characteristics, VNS parameters, and clinical outcomes of enrolled patients.

	**Anagraphics**		**Clinical information**	**VNS parameters**				**Seizures/month**		**Outcome**	
**#°**	**Sex**	**Age**	**Diagnosis**	**Output (mA)**	**Pattern**	**Frequency (Hz)**	**Pulse Width (mcsec)**	**Pre-imp**	**Post-imp**	**Labar**	**Mc Hugh Class**
1	M	45	*Focal epilepsy*	2.00	30″ on, 5′ off	30	500	11.80	11.25	4.66	Class III A
2	F	31	*Lennox-Gastaut S*.	2.00	7″ on, 1.7′ off	25	250	43.70	47.40	−8.47	Class III B
3	M	48	*Focal epilepsy*	1.75	30″ on, 5′ off	30	500	3.00	0,43	85.67	Class I B
4	F	61	*Focal epilepsy*	1.50	30″ on, 5′ off	25	500	20.00	0.10	99.50	Class I A
5	M	42	*Lennox-Gastaut S*.	2.00	30″ on, 5′ off	30	500	4.00	0.67	83.25	Class I B
6	M	46	*Focal epilepsy*	1.50	30″ on, 5′ off	25	500	0.13	0.00	100.00	Class I A
7	F	35	*Focal epilepsy*	1.75	30″ on, 5′ off	25	500	0.33	0.33	0.00	Class III B
8	F	34	*Focal epilepsy*	2.00	30″ on, 5′ off	30	500	10.00	10.00	0.00	Class V
9	M	50	*Lennox-Gastaut S*.	2.00	30″ on, 5′ off	30	250	2.00	2.00	0.00	Class III A
10	M	27	Focal epilepsy*	2.25	30″ on, 5′ off	30	250	33.00	8.40	72.00	Class II A

Labar's Index is the opposite per percentage reduction in seizure frequency. Mc Hugh Classification^+^ identifies five classes of clinical response to VNS therapy, depending on seizure reduction, improvement in seizure severity and postictal state, and response to the use of magnet: • Class I: 80–100% reduction in seizure frequency: Class I A: improved ictal or postictal severity; Class I B: no improvement in ictal or postictal severity. • Class II: 50–79% reduction in seizure frequency: Class II A: improved ictal or postictal severity; Class II B: no improvement in ictal or postictal severity. • Class III: <50% reduction in seizure frequency: Class III A: improved ictal or postictal severity; Class III B: no improvement in ictal or postictal severity. • Class IV: magnet benefit only. • Class V: no improvement.

* Tuberous sclerosis.

^+^McHugh et al. (34).

### PLI

On a global scale, in the responder subjects, the PLI was significantly reduced after VNS in the delta (*p* = 0.04) and gamma (*p* < 0.001) bands, but it increased after VNS in the alpha band (*p* = 0.005). No statistically significant differences were observed in the other bands and the non-responder subjects. However, the gamma band showed an increasing trend in non-responders.

In detail, the analysis of channels grouped by scalp region in the responders showed a decrease for the delta band in the frontal region (*p* = 0.04) and gamma band in the central (*p* = 0.02) and parietal (*p* = 0.02) regions. Conversely, the alpha band showed an increase after VNS in the occipital (*p* < 0.001), parietal (*p* = 0.02), and frontal (*p* = 0.02) regions. No statistically significant difference was observed in non-responders.

**Figure 1:**
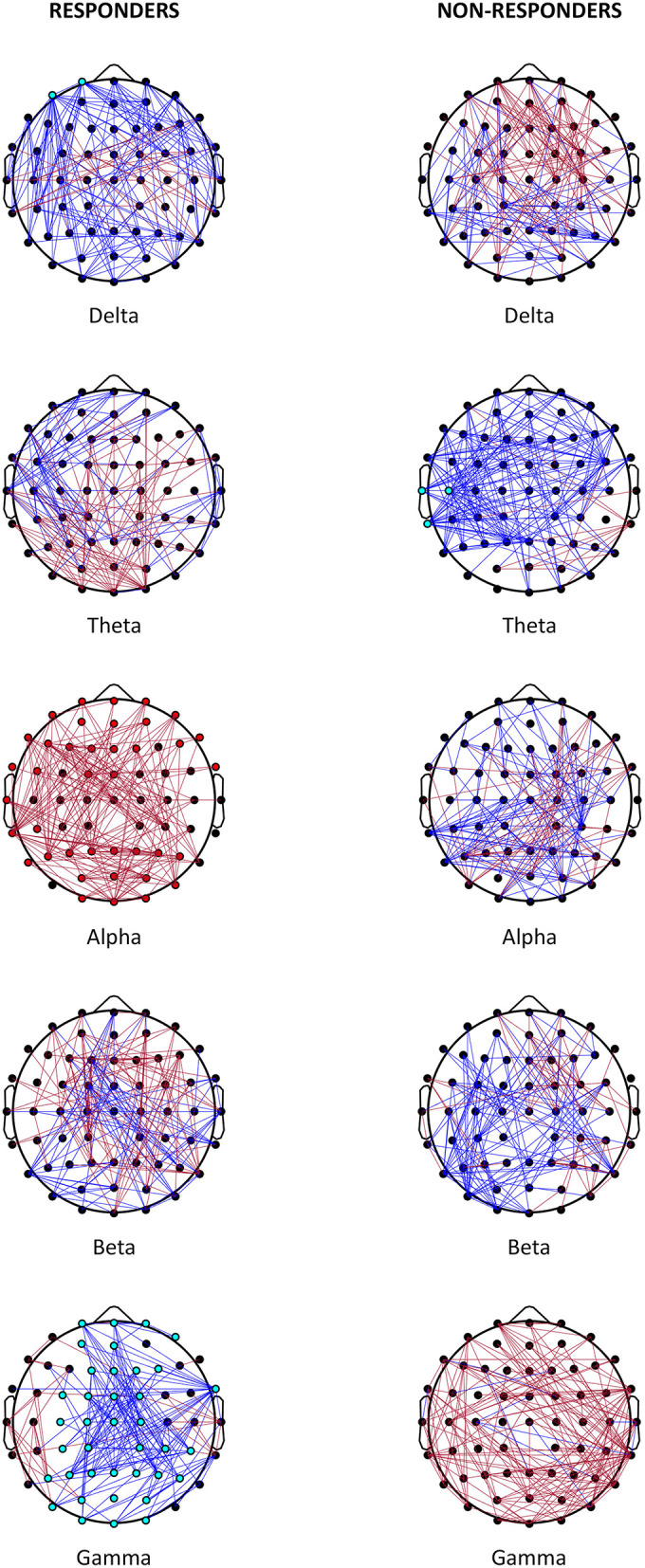
Effect on VNS. Representation of the most relevant post- vs. pre-VNS PLI changes in absolute terms (cut-off: 10%) in responder and non-responder patients. Red lines denote the increased PLI after VNS; blue lines represent the decreased PLI after VNS. Statistically significant channels are represented as red or light blue dots depending on whether the trend increases or decreases.

Table 2 shows the statistically significant results of the analysis for the global scale and single scalp regions. The analysis of individual channels is shown in Figure 1 and Supplementary Table 1. Non-statistically significant results are shown in Supplementary Table 2. **Figure 3** shows a summary of statistically significant changes on a global basis.

**Table 2:** Effects on PLI.

		**Global**		**Frontal**		**Temporal**		**Occipital**		**Parietal**		**Central**	
		***R***	**NR**	***R***	**NR**	***R***	**NR**	***R***	**NR**	***R***	**NR**	***R***	**NR**
Delta	*p*-value	**0.04**	0.98	**0.04**	0.67	0.45	0.67	0.28	0.53	0.30	0.62	0.90	0.90
	trend	**↓**		**↓**									
	μ_pre_ ±σ_pre_	0.18 ±0.04	0.18 ±0.04	0.19 ±0.05	0.18 ±0.05	0.18 ±0.05	0.18 ±0.06	0.19 ±0.07	0.18 ±0.06	0.19 ±0.06	0.19 ±0.06	0.18 ±0.05	0.18 ±0.05
	μ_post_ ±σ_post_	0.17 ±0.03	0.18 ±0.04	0.17 ±0.04	0.19 ±0.06	0.17 ±0.06	0.17 ±0.06	0.17 ±0.06	0.17 ±0.06	0.17 ±0.04	0.17 ±0.04	0.18 ±0.04	0.18 ±0.05
	Cohen's d	0.33	0.02	0.35	0.16	0.16	0.17	0.29	0.13	0.26	0.22	0.06	0.02
Alpha	*p*-value	**0.005**	0.49	**0.02**	0.36	0.22	0.71	**< 0.001**	0.69	**0.02**	0.83	0.30	0.36
	trend	**↑**		**↑**				**↑**		**↑**			
	μ_pre_ ±σ_pre_	0.15 ±0.04	0.17 ±0.06	0.14 ±0.04	0.15 ±0.05	0.14 ±0.04	0.17 ±0.07	0.16 ±0.08	0.20 ±0.10	0.16 ±0.05	0.19 ±0.08	0.15 ±0.04	0.17 ±0.06
	μ_post_ ±σ_post_	0.18 ±0.07	0.17 ±0.06	0.16 ±0.06	0.14 ±0.05	0.16 ±0.06	0.17 ±0.08	0.21 ±0.11	0.19 ±0.10	0.20 ±0.09	0.19 ±0.08	0.17 ±0.06	0.16 ±0.06
	Cohen's d	0.55	0.10	0.47	0.23	0.37	0.03	0.57	0.08	0.51	0.01	0.34	0.20
Gamma	*p*-value	**< 0.001**	0.69	0.06	0.67	0.95	0.71	0.09	0.36	**0.02**	0.36	**0.02**	0.94
	trend	**↓**								**↓**		**↓**	
	μ_pre_ ±σ_pre_	0.08 ±0.02	0.07 ±0.01	0.07 ±0.02	0.07 ±0.01	0.08 ±0.02	0.07 ±0.02	0.08 ±0.03	0.07 ±0.02	0.08 ±0.03	0.07 ±0.01	0.08 ±0.03	0.07 ±0.01
	μ_post_ ±σ_post_	0.07 ±0.01	0.07 ±0.04	0.07 ±0.01	0.07 ±0.04	0.07 ±0.02	0.08 ±0.07	0.07 ±0.02	0.08 ±0.06	0.07 ±0.02	0.08 ±0.05	0.07 ±0.02	0.07 ±0.03
	Cohen's d	0.61	0.17	0.39	0.05	0.11	0.15	0.41	0.34	0.53	0.23	0.47	0.12

Statistically significant results are highlighted in bold. The direction of the arrows represents the trend of change: upward stands for a statistically significant increase and downward stands for a decrease. Non-significant results are reported in the Supplementary Table 2. Statistically significant results of the *U*-test of PLI for the global scale and single scalp regions analyses. Values are reported as *p*-value, μ_pre_ ± σ_pre_ (i.e., mean ± standard deviation before VNS), μ_post_ ± σ_post_ (i.e., mean ± standard deviation after VNS), and Cohen's d (i.e., effect size).

### Aperiodic parameters

The analysis of aperiodic parameters on a global basis showed an opposite behavior in the group of responders and non-responders for the offset and exponent parameters. In responders, a statistically significant reduction was observed in the offset (*p* = 0.02) and exponent (*p* = 0.02) parameters after VNS, but in non-responders, increases in offset (*p* < 0.001) and exponent (*p* = 0.003) parameters were noticed after VNS.

In the analysis of the single regions of the scalp after VNS, the responders showed a reduction of the offset parameter in the frontal (*p* = 0.04), temporal (*p* = 0.005), and occipital (*p* = 0.04) regions and a statistically significant reduction of the exponent parameter in the temporal region (*p* < 0.001).

Conversely, in non-responders, after VNS, a statistically significant increase was recorded in the offset parameter in all scalp regions (*p* ≤ 0.007) and exponent parameter in the frontal (*p* = 0.003), temporal (*p* = 0.003), parietal (*p* = 0.007), and central (*p* = 0.007) regions.

**Figure 2:**
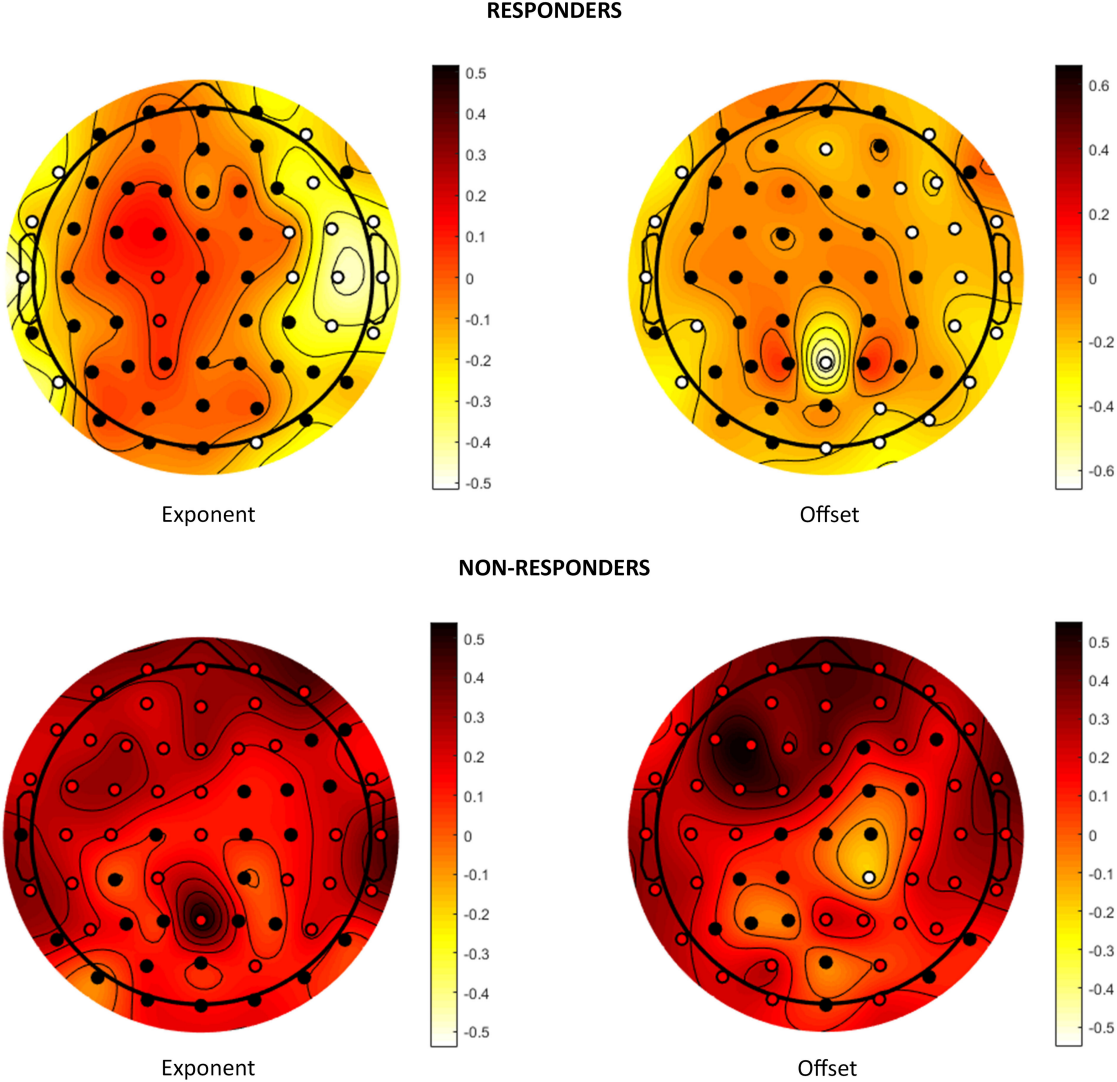
Effects on aperiodic components. Visual representation of the change in the exponent and offset parameters after VNS in responders and non-responders. Colors represent the trend of change: dark red stands for increase after VNS and light yellow for decrease. Statistically significant channels are highlighted as red or white dots depending on whether the trend is increasing or decreasing.

**Figure 3:**
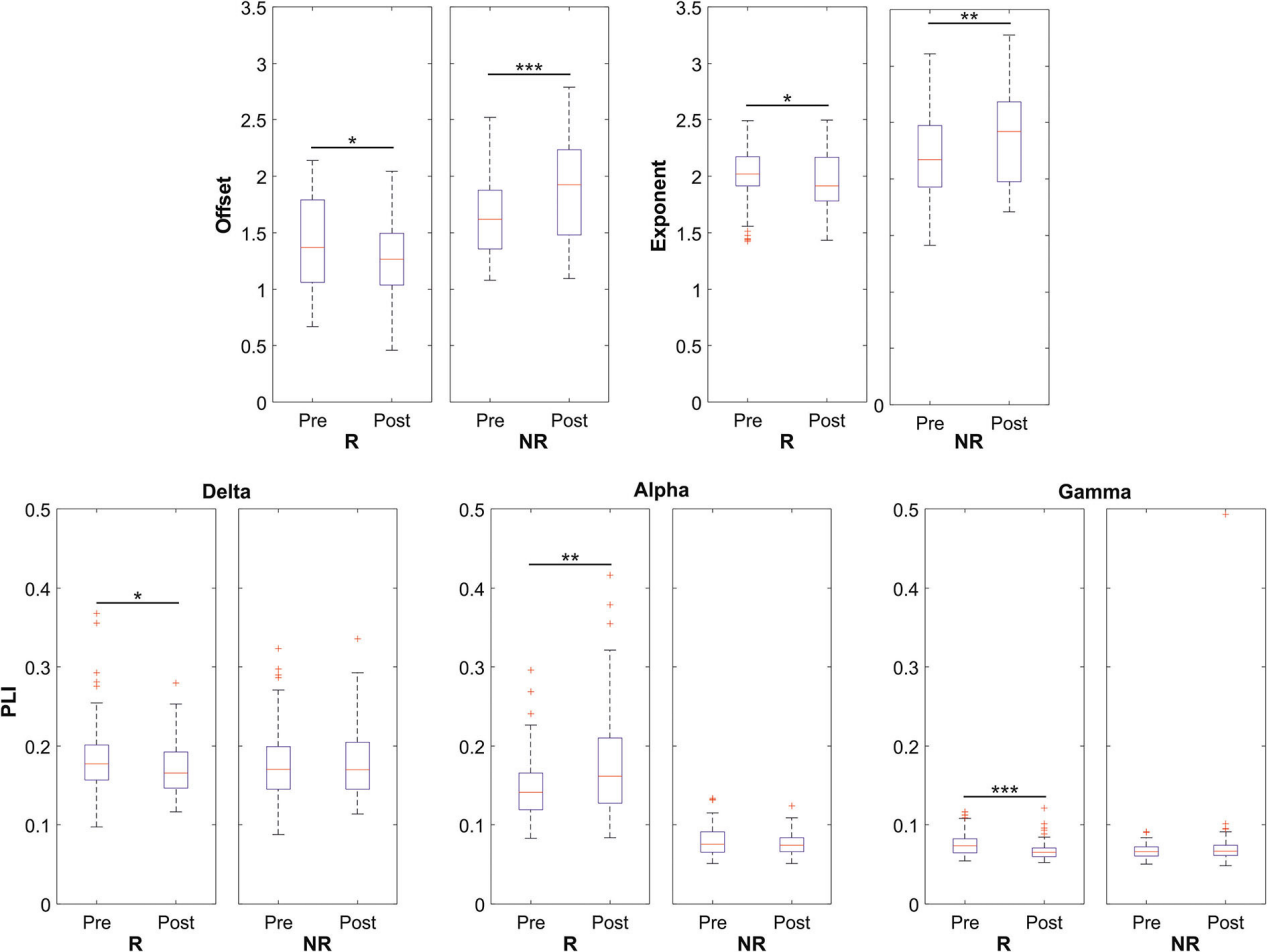
Data distributions of offset, exponent, and PLI parameters for both responders (R) and non-responders (NR) patients in the global scale analysis. Statistically significant distributions are represented by boxplots in terms of median value (red line) and 75th and 25th percentiles (upper and lower limits of the blue box, respectively). In this representation, black whiskers and red crosses identify the distribution extrema and the outliers, respectively. *p < 0.05, **p < 0.01, ***p < 0.001.

The results are shown in Table 3 and graphically depicted in Figure 2. The analysis of individual channels is shown in Figure 2 and Supplementary Table 3. Figure 3 shows a summary of statistically significant changes on a global basis.

**Table 3:** Effects on aperiodic parameters.

		**Global**		**Frontal**		**Temporal**		**Occipital**		**Parietal**		**Central**	
		***R***	**NR**	***R***	**NR**	***R***	**NR**	***R***	**NR**	***R***	**NR**	***R***	**NR**
Offset	*p*-value	**0.02**	**< 0.001**	**0.04**	**0.002**	**0.005**	**< 0.001**	**0.04**	**0.007**	0.07	**< 0.001**	0.06	**0.005**
	trend	**↓**	**↑**	**↓**	**↑**	**↓**	**↑**	**↓**	**↑**		**↑**		**↑**
	μ_pre_ ±σ_pre_	1.4 ±0.4	1.7 ±0.3	1.7 ±0.4	1.9 ±0.4	1.7 ±0.4	1.8 ±0.5	1.3 ±0.4	1.6 ±0.3	1.0 ±0.4	1.2 ±0.3	1.2 ±0.4	1.5 ±0.4
	μ_post_ ±σ_post_	1.3 ±0.4	1.9 ±0.5	1.6 ±0.4	2.2 ±0.5	1.5 ±0.4	2.1 ±0.5	1.2 ±0.4	1.7 ±0.4	0.9 ±0.5	1.5 ±0.4	1.1 ±0.4	1.6 ±0.5
	Cohen's d	0.34	0.57	0.36	0.466	0.49	0.74	0.32	0.47	0.30	0.76	0.29	0.48
Exponent	*p*-value	**0.02**	**0.003**	0.07	**0.003**	**< 0.001**	**0.003**	0.19	0.27	0.19	**0.007**	0.19	**0.007**
	trend	**↓**	**↑**		**↑**	**↓**	**↑**				**↑**		**↑**
	μ_pre_ ±σ_pre_	2.0 ±0.2	2.2 ±0.4	2.0 ±0.3	2.3 ±0.4	1.9 ±0.2	2.1 ±0.5	2.1 ±0.2	2.2 ±0.4	2.0 ±0.2	2.1 ±0.4	2.0 ±0.3	2.2 ±0.5
	μ_post_ ±σ_post_	1.9 ±0.3	2.4 ±0.4	1.9 ±0.3	2.5 ±0.4	1.7 ±0.3	2.3 ±0.5	2.0 ±0.3	2.3 ±0.5	2.0 ±0.3	2.3 ±0.4	2.0 ±0.2	2.4 ±0.4
	Cohen's d	0.23	0.47	0.26	0.497	0.71	0.50	0.10	0.19	0.07	0.50	0.14	0.49

Statistically significant results are highlighted in bold. The direction of the arrows represents the trend of change: upward stands for a statistically significant increase and downward stands for a decrease. Results of the *U*-test of aperiodic parameters on a global scale and single scalp regions (*p*-value, μ_pre_ ± σ_pre_ = mean ± standard deviation before VNS, μ_post_ ± σ_post_ = mean ± standard deviation after VNS, Cohens'd = effect size).

Finally, Spearman's rank correlation coefficient showed that changes in the PLI on a global basis did not significantly correlate with clinical responses. By contrast, both aperiodic parameters demonstrated a negative correlation between the relative variation of the parameter after the VNS implant and Labar's index (*p* = 0.04 for the exponent, *p* =0.01 for the offset). Table 4 shows the results.

**Table 4:** Correlation with clinical response.

	**Exponent**	**Offset**	**PLI**				
			**Delta**	**Theta**	**Alpha**	**Beta**	**Gamma**
*p*-value	**0.04**	**0.01**	0.45	0.85	0.54	0.85	0.45
ρ	−0.64	−0.76	−0.46	0.08	0.35	0.07	−0.47

Statistically significant results are highlighted in bold. Correlation between change in PLI and aperiodic parameters and Labar's index (*p*-value and ρ = Spearman's rank correlation coefficient).

## Discussion

This study evaluated the effect of VNS on Fc and aperiodic EEG parameters of DRE subjects and their correlation with clinical response, by analyzing resting state epochs free of artifact and interictal abnormalities.

We hypothesized that VNS therapy acts with a chronic pathway by modifying the neuronal network and thus induces synaptic plasticity, acting not only on specific frequency bands (analyzed with Fc) but also on the background activity of the brain that is not affected by the environment (aperiodic components) and that this effect is different in responder and non-responder patients.

### PLI

During and before epileptic activity, hyper synchronization occurs in the brain network recorded by EEG, which corresponds to the simultaneous firing of a neural population (38). Starting from this scenario, one hypothesis about the antiepileptic activity of VNS is the desynchronization of the EEG track, which is different in responders and non-responders (39, 40). Previous studies by our group analyzed Fc on 19-channel scalp EEG tracks and demonstrated instead an increase in synchronization and power spectrum for the gamma (17, 18) and theta (16) band and its correlation with better clinical outcomes. Later on, other research showed a correlation between EEG desynchronization and clinical response: Bodin et al. (28) evaluated the influence of VNS, showing that responders had a lower global synchronization (EEG broadband and interictal) than non-responders; Sangare et al. (29) found out that responders had a lower PLI during ON period in delta, theta, and beta bands; Bartolomei et al. (30) stated that subjects responding to VNS had lower synchronization during the ON period than the OFF period on stereotactic EEG recordings; recent research by Lanzone et al. (41) showed a significant and widespread action of VNS cycles on network connectivity, mostly on delta and theta band, which demonstrated a desynchronization in brain activity during and after VNS stimulation. Other research has also shown how action on the neuronal network correlates with clinical response to other forms of neuromodulation and pharmacological therapies (13, 42).

The 19-channel EEG provides a high temporal resolution but a poor spatial resolution for Fc analysis compared with fMRI (8, 9). This limitation can be overcome by increasing the number of EEG channels: for example, 64-channel EEG systems with a high sampling rate (1,024 Hz) improve spatial resolution and reliability (43) of Fc measures while maintaining an excellent temporal resolution and a tolerable quickness montage for subjects with severe pathologies, such as LGS.

The careful selection of epochs, which are free of muscle and environmental artifacts and interictal abnormalities and recorded in wakefulness and resting state, is aimed at the analysis of VNS-induced changes in the EEG background activity, which can represent a mechanism of action of VNS therapy not only on seizure reduction but also on the improvement of vigilance (44) and quality of life (45). From our data, we could also speculate a correlative relationship between the reduction in seizure frequency and changes in brain connectivity, so that VNS leads to seizure reduction and the lack of seizures modifies the brain network: the careful selection of epochs without epileptic abnormalities was intended to reduce the influence of the reduction of these abnormalities on the network analysis.

Based on our results, the PLI of non-responder subjects was not significantly modified by VNS. On the contrary, in the responders, we observed a significant desynchronization of the delta and gamma bands in the frontal and parieto-central regions, respectively, and an increase in the synchronization of the alpha band in the frontal and parieto-occipital region.

A correlation between delta activity and seizure risk has been demonstrated (46), and delta activity is most represented in the EEG of subjects with epilepsy (47). Although recently questioned due to possible muscle influences (36), Ramp et al. (48) and Hughes et al. (49) documented that rapid background activity in the gamma band indicates the presence of an epileptogenic condition and is related to epileptogenesis. Consequently, desynchronization of delta and gamma bands could represent the reduction of periodic epileptiform activity (50), which is already questioned as a possible mechanism of action of VNS (51).

Changes in PLI on the alpha band, especially in the parieto-occipital area, confirmed the well known clinical experience that a reduction in the frequency of seizures corresponds to an increased representation and synchronization of the posterior dominant rhythm, which is usually less evident in subjects with DRE, which instead show a greater occurrence of slow activity (47, 52). This finding demonstrated the action of VNS not only on a specific band but also on the improvement of the general organization of background brain activity.

It has also been shown that in Alzheimer's and autism diseases, there is less representation of alpha rhythm and a higher representation of slow frequencies and this correlates with cognitive impairment (53, 54): consequently, it could be speculated that increased alpha activity and decreased delta activity is a representation of better cognitive performance after VNS.

Our previous study demonstrated a significant change in Fc on the theta band (16), but it was not confirmed by these data; this variability may be due to the different samples analyzed and different analysis techniques (e.g., the use of 64-channel EEG and selection of epochs without distinction of ON and OFF period of VNS).

### Aperiodic parameters

Although Fc analysis gives important information on modifications induced by chronic VNS, the classical subdivision of the EEG signal in narrow frequency bands fails to reflect the whole brain's electrical activity. The existence of an aperiodic 1/f-like activity constitutes the major component of the EEG trace (19) and can influence the analysis of Fc based on conventional frequency bands (26). Moreover, the presence of different bands rapidly varies even in the same individual in different environmental conditions; on the contrary, aperiodic components are highly conserved in individuals (27).

Two parameters are used to define aperiodic components: the offset which has been correlated with the spiking of neuronal population (22) and the exponent with the integration of synaptic currents (55). To the best of our knowledge, no studies on the effects of VNS on aperiodic EEG parameters have been published thus far.

Our results showed that the offset and exponent parameters after VNS had opposite behaviors in responder and non-responder subjects (Figure 2). The responders showed a reduction of these parameters after VNS, but the values increased in non-responders. When examining the different scalp regions, the decrease in responders was evident in the frontotemporal area, and the increase in non-responders was diffused on the whole. The reduction of these parameters in responders after VNS implantation could represent lower neuronal excitability, which did not occur in non-responder subjects and reflected the VNS action in reducing the likelihood of presenting seizures. An increase in the exponent and offset in subjects with schizophrenia compared with healthy subjects and in ADHD subjects not receiving drug therapy compared with treated subjects has been demonstrated (23, 24): the reduction of aperiodic components after VNS could therefore represent an improvement in the neuronal network correlating with the reduction of psychiatric symptoms and improvement in quality of life; on the contrary, clinical worsening due to disease progression could justify the increase in offset and exponent that was recorded in non-responders.

A correlation with Labar's index was found only for the aperiodic parameters and not for the PLI (Table 4). Given that the offset and exponent parameters are highly conserved and exhibit little variability for a given subject (27), their variation can better represent a modification of brain connections, and an improved correlation with clinical response will confirm their central role in understanding the mechanisms underlying the efficacy of VNS.

### Conclusion

Previous studies demonstrated that long-term plasticity is the neurophysiological correlate of changes in the brain network (56); thus, changes in the latter will represent the action of VNS therapy on synaptic plasticity (6).

The results of this study revealed how aperiodic components delineated significant opposite trends while comparing pre- and post-VNS implantation for responders and non-responders, with higher robustness compared with PLI. Moreover, aperiodic component parameters significantly correlated with the clinical effect of VNS, as represented by Labar's index, conversely from PLI.

The use of two different methods (Fc and aperiodic components) allowed us to analyze the action of VNS therapy on neuronal connectivity and thus on synaptic plasticity in two different and complementary ways: on the one hand, the PLI provides information on the action on specific rhythms related to brain activities related to the given task (resting state and mental calculation among other) or pathological activity (epileptic abnormalities) (47). On the other hand, the aperiodic components, strongly preserved and specific fingerprints in each subject (27), provide information about the effect on a more widespread and constant activity. Consequently, the reduction in seizure frequency may affect partially, as shown, the connectivity of individual frequency bands, by reducing synchronization in the delta and gamma bands and increasing synchronization in the alpha band. Furthermore, the change in the aperiodic component does not depend on the change in rhythms but on the change in background activity induced by VNS and is influenced by the effect on seizure frequency, disease progression, and cognitive performance (23, 24). Thus, both methods represent the effect of chronic VNS therapy on synaptic plasticity.

This study was affected by some limitations. Given the small dataset, we were unable to further categorize the subjects depending on their diagnosis (e.g., LGS and focal). However, this study aimed to highlight the action of VNS therapy on the EEG background, regardless of the underlying diagnosis, to understand the mechanisms behind the action regardless of the pathology. Moreover, the observation limit of 1 year was arbitrarily imposed to assess the effects of chronic stimulation. Thus, a longer or shorter observation time can influence the results. Remarkably, we were interested in the exploration of the chronic effect on neuronal connectivity, so epochs were selected regardless of the ON or OFF period, so not consider the acute stimulation effect only (41). In addition, modification of the seizure frequency cannot completely explain the response to VNS. Thus, we emphasize the need to adequately parameterize clinical response to standardize studies.

Although conducted on a small sample, this work paves the way toward new analyses focused on the study of the effect of VNS on EEG aperiodic components. These steps can lead to further insights into the mechanisms of action of VNS therapy on brain networks, its intrinsic components, such as the background activity, and not only on specific frequency bands, thus allowing a better understanding of the action of VNS on the general wellbeing of the subject. The good correlation between these parameters and clinical response suggests that they may be the keys to understanding the clinical efficacy of VNS neuromodulation.

## Data availability statement

The raw data supporting the conclusions of this article will be made available by the authors, without undue reservation.

## Ethics statement

The studies involving human participants were reviewed and approved by Ethics Committee of the AOU Cagliari. The patients/participants provided their written informed consent to participate in this study.

## Author contributions

RC: drafting the manuscript, study concept and design, patient enrollment, acquisition, epoch selection, analysis, and interpretation of data. SL: drafting the manuscript, creation of Athena, a toolbox for extraction and analysis of Fc and aperiodic measures and statistical analysis, and analysis and interpretation of data. GB and DP: creation of an ad-hoc graphical interface for data import, epoch selection, and signal processing and critical revision of the manuscript for important intellectual content. LP: patient enrollment and critical revision of the manuscript for important intellectual content. GP and CC: surgical procedure and critical revision of the manuscript for important intellectual content. GD: critical revision of the manuscript for important intellectual content. MP: study supervision, study concept and design patient enrollment, epoch selection, interpretation of data, referent for ethical procedure, and critical revision of the manuscript for important intellectual content. All authors approved the final manuscript.

## Funding

This work was supported by internal funds through the University of Cagliari.

## Conflict of interest

Unrelated to this study, in the past 3 years, Author RC received support for congress attendance from a distributor of VNS therapy and Author MP had a consulting contract with Livanova, USA. The remaining authors declare that the research was conducted in the absence of any commercial or financial relationships that could be construed as a potential conflict of interest.

## Publisher's note

All claims expressed in this article are solely those of the authors and do not necessarily represent those of their affiliated organizations, or those of the publisher, the editors and the reviewers. Any product that may be evaluated in this article, or claim that may be made by its manufacturer, is not guaranteed or endorsed by the publisher.
