# Supplementary material for: Estimated EEG functional connectivity and aperiodic component induced by vagal nerve stimulation in patients with drug-resistant epilepsy
Source: Front Neurol. 2022 Nov 23;13:1030118. doi: 10.3389/fneur.2022.1030118 (PMC9728998; doi:10.3389/fneur.2022.1030118)
Supplement: Supplementary file 1 [file Table_1.docx]

**Supplementary Table 1:** Results of u test of PLI for single-channel analyses. Values are reported as p-value, μ_pre_ ± σ_pre_ (i.e., mean ± standard deviation before VNS), μ_post_ ± σ_post_ (i.e., mean ± standard deviation after VNS), and Cohen’s d (i.e., effect size). R = responders; NR = non-responders.

Statistically significant results are highlighted in bold. The direction of arrows represents the trend of change: upward stands for a statistically significant increase and downward for a decrease.

|  |  | *Delta* | | *Theta* | | *Alpha* | | *Beta* | | *Gamma* | |
| --- | --- | --- | --- | --- | --- | --- | --- | --- | --- | --- | --- |
|  |  | *R* | *NR* | *R* | *NR* | *R* | *NR* | *R* | *NR* | *R* | *NR* |
| FP1 | *p*-value | **< 0.001** | 0.84 | 0.52 | 0.87 | **0.01** | 0.34 | 0.95 | 0.93 | **0.01** | 0.82 |
|  | trend | **↓** |  |  |  | **↑** |  |  |  | **↓** |  |
|  | μ_pre_  ± σ_pre_ | 0.20  ±0.06 | 0.18  ±0.05 | 0.13  ±0.04 | 0.14  ±0.06 | 0.14  ±0.05 | 0.17  ±0.06 | 0.07  ±0.02 | 0.08  ±0.02 | 0.074  ±0.021 | 0.06  ±0.02 |
|  | μ_post_  ± σ_post_ | 0.17  ±0.06 | 0.18  ±0.06 | 0.13  ±0.04 | 0.13  ±0.05 | 0.18  ±0.07 | 0.15  ±0.07 | 0.07  ±0.02 | 0.07  ±0.02 | 0.065  ±0.019 | 0.07  ±0.05 |
|  | Cohen’s d | 0.58 | 0.13 | 0.19 | 0.14 | 0.50 | 0.25 | 0.02 | 0.06 | 0.442 | 0.16 |
| AF7 | *p*-value | **< 0.1** | 0.93 | 0.76 | 0.69 | **< 0.001** | 0.69 | 0.86 | 0.85 | 0.52 | 0.93 |
|  | trend | **↓** |  |  |  | **↑** |  |  |  |  |  |
|  | μ_pre_  ± σ_pre_ | 0.20  ±0.07 | 0.18  ±0.05 | 0.13  ±0.05 | 0.14  ±0.05 | 0.14  ±0.05 | 0.17  ±0.06 | 0.07  ±0.02 | 0.08  ±0.02 | 0.068  ±0.018 | 0.07  ±0.02 |
|  | μ_post_  ± σ_post_ | 0.17  ±0.05 | 0.18  ±0.06 | 0.12  ±0.04 | 0.13  ±0.05 | 0.17  ±0.07 | 0.16  ±0.06 | 0.07  ±0.02 | 0.07  ±0.02 | 0.066  ±0.017 | 0.07  ±0.04 |
|  | Cohen’s d | 0.61 | 0.01 | 0.14 | 0.2 | 0.58 | 0.19 | 0.01 | 0.14 | 0.129 | 0.13 |
| AF3 | *p*-value | 0.07 | 0.92 | 0.83 | 0.80 | **0.007** | 0.54 | 0.77 | 0.73 | **0.03** | 0.92 |
|  | trend |  |  |  |  | **↑** |  |  |  | **↓** |  |
|  | μ_pre_  ± σ_pre_ | 0.19  ±0.06 | 0.18  ±0.06 | 0.13  ±0.04 | 0.14  ±0.05 | 0.14  ±0.05 | 0.17  ±0.06 | 0.07  ±0.02 | 0.08  ±0.03 | 0.073  ±0.021 | 0.07  ±0.02 |
|  | μ_post_  ± σ_post_ | 0.17  ±0.05 | 0.18  ±0.06 | 0.13  ±0.05 | 0.13  ±0.05 | 0.18  ±0.07 | 0.16  ±0.07 | 0.07  ±0.02 | 0.07  ±0.02 | 0.065  ±0.018 | 0.07  ±0.05 |
|  | Cohen’s d | 0.35 | 0.08 | 0.04 | 0.19 | 0.53 | 0.19 | 0.1 | 0.26 | 0.406 | 0.1 |
| F7 | *p*-value | 0.78 | 0.92 | 0.55 | 0.36 | **0.01** | 0.73 | 0.61 | 0.89 | 0.92 | 0.98 |
|  | trend |  |  |  |  | **↑** |  |  |  |  |  |
|  | μ_pre_  ± σ_pre_ | 0.18  ±0.07 | 0.17  ±0.06 | 0.13  ±0.05 | 0.14  ±0.05 | 0.13  ±0.05 | 0.17  ±0.06 | 0.07  ±0.02 | 0.07  ±0.02 | 0.068  ±0.019 | 0.07  ±0.02 |
|  | μ_post_  ± σ_post_ | 0.17  ±0.05 | 0.18  ±0.07 | 0.12  ±0.04 | 0.12  ±0.05 | 0.17  ±0.07 | 0.16  ±0.06 | 0.07  ±0.02 | 0.07  ±0.02 | 0.068  ±0.017 | 0.07  ±0.05 |
|  | Cohen’s d | 0.14 | 0.10 | 0.19 | 0.26 | 0.53 | 0.16 | 0.07 | 0.14 | 0.009 | 0.12 |
| F5 | *p*-value | 0.14 | 0.93 | 0.78 | 0.32 | **< 0.001** | 0.71 | 0.96 | 0.47 | 0.21 | 0.98 |
|  | trend |  |  |  |  | **↑** |  |  |  |  |  |
|  | μ_pre_  ± σ_pre_ | 0.19  ±0.06 | 0.18  ±0.07 | 0.13  ±0.05 | 0.14  ±0.06 | 0.13  ±0.05 | 0.17  ±0.06 | 0.07  ±0.02 | 0.08  ±0.03 | 0.07  ±0.02 | 0.07  ±0.02 |
|  | μ_post_  ± σ_post_ | 0.18  ±0.05 | 0.18  ±0.06 | 0.12  ±0.04 | 0.13  ±0.05 | 0.17  ±0.07 | 0.16  ±0.06 | 0.07  ±0.02 | 0.07  ±0.02 | 0.07  ±0.02 | 0.07  ±0.05 |
|  | Cohen’s d | 0.24 | 0.04 | 0.14 | 0.32 | 0.61 | 0.16 | 0.05 | 0.35 | 0.26 | 0.1 |
| F3 | *p*-value | 0.86 | 0.73 | 0.63 | 0.95 | **< 0.001** | 0.82 | 0.94 | 0.32 | 0.53 | 0.64 |
|  | trend |  |  |  |  | **↑** |  |  |  |  |  |
|  | μ_pre_  ± σ_pre_ | 0.18  ±0.06 | 0.19  ±0.07 | 0.13  ±0.05 | 0.14  ±0.06 | 0.14  ±0.05 | 0.17  ±0.07 | 0.07  ±0.02 | 0.08  ±0.03 | 0.07  ±0.02 | 0.07  ±0.02 |
|  | μ_post_  ± σ_post_ | 0.18  ±0.05 | 0.18  ±0.06 | 0.13  ±0.04 | 0.14  ±0.05 | 0.17  ±0.07 | 0.16  ±0.07 | 0.07  ±0.02 | 0.08  ±0.02 | 0.07  ±0.02 | 0.07  ±0.04 |
|  | Cohen’s d | 0.03 | 0.19 | 0.08 | 0.04 | 0.65 | 0.13 | 0.05 | 0.38 | 0.20 | 0.04 |
| F1 | *p*-value | 0.83 | 0.86 | 0.39 | 0.88 | **0.007** | 0.87 | 0.89 | 0.94 | **0.007** | 0.97 |
|  | trend |  |  |  |  | **↑** |  |  |  | **↓** |  |
|  | μ_pre_  ± σ_pre_ | 0.180  ±0.06 | 0.19  ±0.07 | 0.13  ±0.05 | 0.15  ±0.06 | 0.15  ±0.05 | 0.17  ±0.07 | 0.08  ±0.02 | 0.08  ±0.03 | 0.08  ±0.02 | 0.07  ±0.02 |
|  | μ_post_  ± σ_post_ | 0.17  ±0.05 | 0.18  ±0.06 | 0.14  ±0.05 | 0.14  ±0.05 | 0.18  ±0.07 | 0.16  ±0.07 | 0.08  ±0.02 | 0.08  ±0.02 | 0.07  ±0.02 | 0.07  ±0.04 |
|  | Cohen’s d | 0.11 | 0.15 | 0.2 | 0.12 | 0.54 | 0.1 | 0.00 | 0.11 | 0.49 | 0.05 |
| FT7 | *p*-value | 0.59 | 0.93 | 0.52 | 0.28 | 0.08 | 0.92 | 0.96 | 0.93 | 0.27 | 0.98 |
|  | trend |  |  |  |  |  |  |  |  |  |  |
|  | μ_pre_  ± σ_pre_ | 0.19  ±0.07 | 0.17  ±0.07 | 0.13  ±0.05 | 0.15  ±0.06 | 0.13  ±0.04 | 0.16  ±0.07 | 0.07  ±0.02 | 0.07  ±0.02 | 0.07  ±0.02 | 0.07  ±0.02 |
|  | μ_post_  ± σ_post_ | 0.18  ±0.06 | 0.18  ±0.07 | 0.12  ±0.04 | 0.12  ±0.05 | 0.16  ±0.07 | 0.16  ±0.06 | 0.07  ±0.03 | 0.07  ±0.03 | 0.07  ±0.02 | 0.07  ±0.06 |
|  | Cohen’s d | 0.16 | 0.02 | 0.2 | 0.3758 | 0.45 | 0.08 | 0.03 | 0.08 | 0.17 | 0.12 |
| FC5 | *p*-value | 0.34 | 0.93 | 0.08 | 0.47 | 0.06 | 0.84 | 0.86 | 0.71 | 0.64 | 0.86 |
|  | trend |  |  |  |  |  |  |  |  |  |  |
|  | μ_pre_  ± σ_pre_ | 0.18  ±0.07 | 0.18  ±0.07 | 0.13  ±0.05 | 0.14  ±0.06 | 0.13  ±0.05 | 0.16  ±0.06 | 0.07  ±0.02 | 0.08  ±0.03 | 0.07  ±0.02 | 0.07  ±0.02 |
|  | μ_post_  ± σ_post_ | 0.17  ±0.05 | 0.18  ±0.07 | 0.11  ±0.04 | 0.13  ±0.05 | 0.16  ±0.07 | 0.15  ±0.06 | 0.07  ±0.02 | 0.07  ±0.02 | 0.07  ±0.02 | 0.07  ±0.06 |
|  | Cohen’s d | 0.27 | 0.05 | 0.36 | 0.25 | 0.44 | 0.14 | 0.01 | 0.25 | 0.07 | 0.08 |
| FC3 | *p*-value | 0.49 | 0.95 | 1.00 | 0.92 | 0.13 | 0.82 | 0.84 | 0.22 | **0.007** | 0.86 |
|  | trend |  |  |  |  |  |  |  |  | **↓** |  |
|  | μ_pre_  ± σ_pre_ | 0.18  ±0.05 | 0.18  ±0.06 | 0.13  ±0.05 | 0.14  ±0.06 | 0.14  ±0.05 | 0.16  ±0.07 | 0.08  ±0.02 | 0.09  ±0.03 | 0.07  ±0.02 | 0.07  ±0.02 |
|  | μ_post_  ± σ_post_ | 0.17  ±0.06 | 0.18  ±0.07 | 0.13  ±0.05 | 0.13  ±0.05 | 0.17  ±0.08 | 0.15  ±0.06 | 0.08  ±0.03 | 0.08  ±0.02 | 0.07  ±0.02 | 0.07  ±0.04 |
|  | Cohen’s d | 0.11 | 0.06 | 0.01 | 0.09 | 0.4 | 0.16 | 0.02 | 0.45 | 0.41 | 0.05 |
| FC1 | *p*-value | 0.59 | 0.93 | 0.66 | 0.82 | **0.007** | 0.73 | 0.59 | 0.69 | **< 0.001** | 0.94 |
|  | trend |  |  |  |  | **↑** |  |  |  | **↓** |  |
|  | μ_pre_  ± σ_pre_ | 0.18  ±0.06 | 0.19  ±0.07 | 0.13  ±0.05 | 0.15  ±0.06 | 0.15  ±0.05 | 0.18  ±0.09 | 0.08  ±0.02 | 0.08  ±0.03 | 0.08  ±0.02 | 0.07  ±0.02 |
|  | μ_post_  ± σ_post_ | 0.17  ±0.05 | 0.18  ±0.07 | 0.14  ±0.06 | 0.14  ±0.06 | 0.18  ±0.08 | 0.16  ±0.08 | 0.08  ±0.03 | 0.08  ±0.03 | 0.07  ±0.02 | 0.07  ±0.03 |
|  | Cohen’s d | 0.13 | 0.05 | 0.16 | 0.16 | 0.54 | 0.21 | 0.04 | 0.2 | 0.51 | 0.07 |
| T3 | *p*-value | 1.00 | 0.86 | 0.52 | 0.15 | 0.07 | 0.98 | 0.96 | 0.77 | 0.78 | 0.93 |
|  | trend |  |  |  |  |  |  |  |  |  |  |
|  | μ_pre_  ± σ_pre_ | 0.18  ±0.07 | 0.18  ±0.07 | 0.12  ±0.05 | 0.15  ±0.06 | 0.13  ±0.04 | 0.16  ±0.07 | 0.07  ±0.02 | 0.07  ±0.02 | 0.07  ±0.02 | 0.07  ±0.02 |
|  | μ_post_  ± σ_post_ | 0.18  ±0.06 | 0.17  ±0.07 | 0.11  ±0.04 | 0.12  ±0.05 | 0.16  ±0.07 | 0.16  ±0.06 | 0.07  ±0.02 | 0.07  ±0.03 | 0.07  ±0.02 | 0.07  ±0.07 |
|  | Cohen’s d | 0.04 | 0.12 | 0.19 | 0.45 | 0.48 | 0.01 | 0.06 | 0.11 | 0.14 | 0.11 |
| C5 | *p*-value | 0.93 | 0.85 | 0.15 | 0.15 | 0.60 | 0.73 | 0.83 | 0.71 | 0.52 | 0.98 |
|  | trend |  |  |  |  |  |  |  |  |  |  |
|  | μ_pre_  ± σ_pre_ | 0.18  ±0.06 | 0.18  ±0.06 | 0.13  ±0.05 | 0.15  ±0.07 | 0.13  ±0.05 | 0.17  ±0.07 | 0.07  ±0.02 | 0.08  ±0.03 | 0.07  ±0.02 | 0.07  ±0.02 |
|  | μ_post_  ± σ_post_ | 0.17  ±0.05 | 0.17  ±0.06 | 0.12  ±0.05 | 0.12  ±0.05 | 0.15  ±0.07 | 0.15  ±0.06 | 0.07  ±0.02 | 0.07  ±0.02 | 0.07  ±0.02 | 0.07  ±0.07 |
|  | Cohen’s d | 0.08 | 0.09 | 0.25 | 0.5 | 0.26 | 0.21 | 0.09 | 0.24 | 0.05 | 0.14 |
| C3 | *p*-value | 0.24 | 0.9 | 0.62 | 0.93 | 0.40 | 0.47 | 0.96 | 0.34 | 0.06 | 0.73 |
|  | trend |  |  |  |  |  |  |  |  |  |  |
|  | μ_pre_  ± σ_pre_ | 0.18  ±0.06 | 0.19  ±0.08 | 0.13  ±0.05 | 0.14  ±0.06 | 0.15  ±0.06 | 0.17  ±0.08 | 0.07  ±0.02 | 0.09  ±0.03 | 0.07  ±0.02 | 0.07  ±0.02 |
|  | μ_post_  ± σ_post_ | 0.17  ±0.06 | 0.18  ±0.06 | 0.12  ±0.05 | 0.13  ±0.05 | 0.17  ±0.09 | 0.14  ±0.05 | 0.07  ±0.03 | 0.08  ±0.03 | 0.07  ±0.02 | 0.07  ±0.04 |
|  | Cohen’s d | 0.2 | 0.18 | 0.12 | 0.11 | 0.32 | 0.39 | 0.05 | 0.37 | 0.32 | 0.06 |
| C1 | *p*-value | 0.73 | 0.73 | 0.83 | 0.92 | 0.22 | 0.39 | 0.83 | 0.68 | **< 0.001** | 0.92 |
|  | trend |  |  |  |  |  |  |  |  | **↓** |  |
|  | μ_pre_  ± σ_pre_ | 0.18  ±0.07 | 0.18  ±0.07 | 0.13  ±0.05 | 0.14  ±0.06 | 0.16  ±0.07 | 0.18  ±0.08 | 0.07  ±0.02 | 0.08  ±0.03 | 0.08  ±0.03 | 0.07  ±0.02 |
|  | μ_post_  ± σ_post_ | 0.17  ±0.05 | 0.17  ±0.06 | 0.12  ±0.05 | 0.14  ±0.06 | 0.19  ±0.1 | 0.16  ±0.09 | 0.08  ±0.03 | 0.08  ±0.03 | 0.07  ±0.02 | 0.07  ±0.03 |
|  | Cohen’s d | 0.14 | 0.21 | 0.03 | 0.06 | 0.35 | 0.21 | 0.1 | 0.22 | 0.57 | 0.17 |
| TP7 | *p*-value | 0.83 | 0.69 | 0.88 | 0.15 | **< 0.001** | 0.64 | 0.74 | 0.77 | 0.44 | 0.69 |
|  | trend |  |  |  |  | **↑** |  |  |  |  |  |
|  | μ_pre_  ± σ_pre_ | 0.18  ±0.07 | 0.18  ±0.06 | 0.12  ±0.04 | 0.15  ±0.06 | 0.12  ±0.04 | 0.2  ±0.12 | 0.07  ±0.02 | 0.08  ±0.03 | 0.07  ±0.02 | 0.07  ±0.02 |
|  | μ_post_  ± σ_post_ | 0.18  ±0.05 | 0.16  ±0.06 | 0.12  ±0.04 | 0.12  ±0.05 | 0.17  ±0.08 | 0.17  ±0.08 | 0.07  ±0.02 | 0.07  ±0.02 | 0.08  ±0.03 | 0.08  ±0.07 |
|  | Cohen’s d | 0.102 | 0.22 | 0.02 | 0.51 | 0.69 | 0.31 | 0.01 | 0.18 | 0.24 | 0.20 |
| CP5 | *p*-value | 0.09 | 0.93 | 0.84 | 0.36 | **0.04** | 0.54 | 0.34 | 0.36 | 0.20 | 0.92 |
|  | trend |  |  |  |  | **↑** |  |  |  |  |  |
|  | μ_pre_  ± σ_pre_ | 0.18  ±0.06 | 0.18  ±0.07 | 0.12  ±0.04 | 0.14  ±0.06 | 0.13  ±0.05 | 0.18  ±0.07 | 0.07  ±0.02 | 0.09  ±0.04 | 0.07  ±0.02 | 0.07  ±0.02 |
|  | μ_post_  ± σ_post_ | 0.17  ±0.05 | 0.17  ±0.06 | 0.12  ±0.05 | 0.13  ±0.05 | 0.16  ±0.06 | 0.16  ±0.08 | 0.07  ±0.02 | 0.08  ±0.02 | 0.07  ±0.02 | 0.08  ±0.07 |
|  | Cohen’s d | 0.33 | 0.09 | 0.07 | 0.35 | 0.45 | 0.19 | 0.20 | 0.35 | 0.24 | 0.13 |
| CP3 | *p*-value | 0.33 | 0.86 | 0.58 | 0.71 | 0.20 | 0.33 | 0.83 | 0.20 | 0.08 | 0.73 |
|  | trend |  |  |  |  |  |  |  |  |  |  |
|  | μ_pre_  ± σ_pre_ | 0.18  ±0.06 | 0.18  ±0.07 | 0.13  ±0.05 | 0.14  ±0.06 | 0.16  ±0.07 | 0.17  ±0.08 | 0.07  ±0.02 | 0.09  ±0.03 | 0.07  ±0.02 | 0.07  ±0.02 |
|  | μ_post_  ± σ_post_ | 0.16 | 0.16  ±0.06 | 0.12  ±0.05 | 0.13  ±0.05 | 0.19  ±0.1 | 0.14  ±0.06 | 0.07  ±0.02 | 0.07  ±0.02 | 0.07  ±0.02 | 0.07  ±0.05 |
|  |  | ±0.05 |  |  |  |  |  |  |  |  |  |
|  | Cohen’s d | 0.23 | 0.2 | 0.10 | 0.24 | 0.35 | 0.37 | 0.02 | 0.45 | 0.33 | 0.03 |
| CP1 | *p*-value | 0.63 | 0.69 | 0.96 | 0.86 | 0.42 | 0.62 | 0.68 | 0.92 | **< 0.001** | 0.62 |
|  | trend |  |  |  |  |  |  |  |  | **↓** |  |
|  | μ_pre_  ± σ_pre_ | 0.18  ±0.06 | 0.19  ±0.08 | 0.13  ±0.05 | 0.15  ±0.06 | 0.17  ±0.076 | 0.19  ±0.1 | 0.07  ±0.02 | 0.08  ±0.03 | 0.08  ±0.03 | 0.07  ±0.02 |
|  | μ_post_  ± σ_post_ | 0.18  ±0.06 | 0.17  ±0.05 | 0.13  ±0.05 | 0.14  ±0.06 | 0.2  ±0.10 | 0.17  ±0.09 | 0.07  ±0.02 | 0.07  ±0.03 | 0.07  ±0.02 | 0.07  ±0.03 |
|  | Cohen’s d | 0.12 | 0.30 | 0.00 | 0.14 | 0.26 | 0.21 | 0.13 | 0.12 | 0.68 | 0.26 |
| T5 | *p*-value | 0.17 | 0.69 | 0.83 | 0.28 | 0.03 | 0.86 | **0.02** | 0.69 | 0.16 | 0.86 |
|  | trend |  |  |  |  |  |  | **↓** |  |  |  |
|  | μ_pre_  ± σ_pre_ | 0.19  ±0.07 | 0.17  ±0.06 | 0.13  ±0.04 | 0.15  ±0.06 | 0.15  ±0.06 | 0.19  ±0.08 | 0.08  ±0.02 | 0.08  ±0.03 | 0.07  ±0.02 | 0.07  ±0.02 |
|  | μ_post_  ± σ_post_ | 0.17  ±0.06 | 0.16  ±0.06 | 0.12  ±0.04 | 0.13  ±0.04 | 0.18  ±0.09 | 0.19  ±0.10 | 0.07  ±0.02 | 0.07  ±0.03 | 0.07  ±0.02 | 0.08  ±0.06 |
|  | Cohen’s d | 0.24 | 0.2 | 0.11 | 0.40 | 0.47 | 0.03 | 0.46 | 0.29 | 0.29 | 0.22 |
| P5 | *p*-value | 0.09 | 0.74 | 0.83 | 0.32 | **< 0.001** | 0.93 | 0.52 | 0.84 | 0.06 | 0.95 |
|  | trend |  |  |  |  | **↑** |  |  |  |  |  |
|  | μ_pre_  ± σ_pre_ | 0.19  ±0.07 | 0.17  ±0.06 | 0.12  ±0.04 | 0.14  ±0.05 | 0.15  ±0.05 | 0.18  ±0.07 | 0.07  ±0.02 | 0.09  ±0.04 | 0.08  ±0.02 | 0.07  ±0.02 |
|  | μ_post_  ± σ_post_ | 0.17  ±0.05 | 0.16  ±0.06 | 0.13  ±0.05 | 0.13  ±0.04 | 0.18  ±0.07 | 0.18  ±0.1 | 0.07  ±0.02 | 0.08  ±0.03 | 0.07  ±0.02 | 0.07  ±0.06 |
|  | Cohen’s d | 0.34 | 0.19 | 0.1 | 0.35 | 0.61 | 0.08 | 0.22 | 0.22 | 0.31 | 0.15 |
| P3 | *p*-value | 0.47 | 0.98 | 0.40 | 0.73 | **0.02** | 0.98 | 0.97 | 0.93 | **< 0.001** | 0.62 |
|  | trend |  |  |  |  | **↑** |  |  |  | **↓** |  |
|  | μ_pre_  ± σ_pre_ | 0.18  ±0.06 | 0.17  ±0.07 | 0.13  ±0.05 | 0.14  ±0.05 | 0.16  ±0.07 | 0.16  ±0.06 | 0.07  ±0.02 | 0.08  ±0.03 | 0.08  ±0.02 | 0.07  ±0.02 |
|  | μ_post_  ± σ_post_ | 0.17  ±0.05 | 0.17  ±0.06 | 0.13  ±0.05 | 0.13  ±0.04 | 0.2  ±0.09 | 0.16  ±0.07 | 0.07  ±0.02 | 0.08  ±0.03 | 0.07  ±0.02 | 0.08  ±0.05 |
|  | Cohen’s d | 0.2 | 0.09 | 0.12 | 0.24 | 0.47 | 0.08 | 0.03 | 0.06 | 0.55 | 0.23 |
| P1 | *p*-value | 0.15 | 1.00 | 0.30 | 0.36 | **0.007** | 0.92 | 0.59 | 0.98 | **< 0.001** | 0.92 |
|  | trend |  |  |  |  | **↑** |  |  |  | **↓** |  |
|  | μ_pre_  ± σ_pre_ | 0.19  ±0.06 | 0.18  ±0.08 | 0.14  ±0.05 | 0.16  ±0.06 | 0.16  ±0.07 | 0.17  ±0.07 | 0.07  ±0.02 | 0.08  ±0.03 | 0.08  ±0.02 | 0.07  ±0.02 |
|  | μ_post_  ± σ_post_ | 0.17  ±0.05 | 0.17  ±0.06 | 0.13  ±0.05 | 0.14  ±0.05 | 0.21  ±0.09 | 0.17  ±0.09 | 0.07  ±0.02 | 0.08  ±0.03 | 0.07  ±0.02 | 0.07  ±0.04 |
|  | Cohen’s d | 0.28 | 0.10 | 0.18 | 0.33 | 0.5 | 0.03 | 0.12 | 0.08 | 0.67 | 0.18 |
| PO7 | *p*-value | 0.17 | 0.84 | 0.72 | 0.64 | 0.21 | 0.89 | 0.13 | 0.15 | **0.01** | 0.73 |
|  | trend |  |  |  |  |  |  |  |  | **↓** |  |
|  | μ_pre_  ± σ_pre_ | 0.19  ±0.06 | 0.17  ±0.06 | 0.12  ±0.04 | 0.14  ±0.05 | 0.16  ±0.06 | 0.17  ±0.08 | 0.08  ±0.02 | 0.09  ±0.04 | 0.08  ±0.02 | 0.06  ±0.02 |
|  | μ_post_  ± σ_post_ | 0.17  ±0.05 | 0.17  ±0.06 | 0.12  ±0.04 | 0.13  ±0.04 | 0.18  ±0.09 | 0.19  ±0.10 | 0.07  ±0.02 | 0.07  ±0.03 | 0.07  ±0.02 | 0.07  ±0.05 |
|  | Cohen’s d | 0.26 | 0.14 | 0.06 | 0.27 | 0.36 | 0.16 | 0.31 | 0.47 | 0.45 | 0.23 |
| PO3 | *p*-value | 0.37 | 0.98 | 0.43 | 0.92 | **0.007** | 1.00 | 0.72 | 0.95 | **< 0.001** | 0.60 |
|  | trend |  |  |  |  | **↑** |  |  |  | **↓** |  |
|  | μ_pre_  ± σ_pre_ | 0.18  ±0.06 | 0.18  ±0.07 | 0.13  ±0.04 | 0.14  ±0.05 | 0.16  ±0.06 | 0.18  ±0.09 | 0.07  ±0.02 | 0.08  ±0.03 | 0.08  ±0.02 | 0.07  ±0.01 |
|  | μ_post_  ± σ_post_ | 0.17  ±0.04 | 0.18  ±0.07 | 0.12  ±0.04 | 0.14  ±0.05 | 0.2  ±0.08 | 0.17  ±0.07 | 0.07  ±0.02 | 0.08  ±0.02 | 0.07  ±0.02 | 0.07  ±0.05 |
|  | Cohen’s d | 0.28 | 0.03 | 0.18 | 0.09 | 0.56 | 0.07 | 0.12 | 0.11 | 0.51 | 0.23 |
| O1 | *p*-value | 0.33 | 0.96 | 0.47 | 0.90 | 0.08 | 0.94 | 0.28 | 0.28 | **< 0.001** | 0.84 |
|  | trend |  |  |  |  |  |  |  |  | **↓** |  |
|  | μ_pre_  ± σ_pre_ | 0.18  ±0.06 | 0.17  ±0.06 | 0.12  ±0.04 | 0.14  ±0.05 | 0.16  ±0.06 | 0.17  ±0.07 | 0.08  ±0.02 | 0.08  ±0.03 | 0.08  ±0.02 | 0.07  ±0.02 |
|  | μ_post_  ± σ_post_ | 0.17  ±0.05 | 0.17  ±0.06 | 0.13  ±0.05 | 0.13  ±0.05 | 0.19  ±0.09 | 0.17  ±0.07 | 0.07  ±0.02 | 0.07  ±0.023 | 0.07  ±0.02 | 0.07  ±0.05 |
|  | Cohen’s d | 0.21 | 0.03 | 0.23 | 0.12 | 0.46 | 0.06 | 0.22 | 0.39 | 0.53 | 0.17 |
| FPZ | *p*-value | **0.02** | 0.95 | 0.57 | 0.78 | **0.02** | 0.47 | 0.91 | 0.85 | **< 0.001** | 0.95 |
|  | trend | **↓** |  |  |  | **↑** |  |  |  | **↓** |  |
|  | μ_pre_  ± σ_pre_ | 0.19  ±0.06 | 0.18  ±0.05 | 0.13  ±0.04 | 0.14  ±0.06 | 0.15  ±0.05 | 0.17  ±0.07 | 0.07  ±0.03 | 0.08  ±0.02 | 0.08  ±0.02 | 0.07  ±0.02 |
|  | μ_post_  ± σ_post_ | 0.17  ±0.06 | 0.18  ±0.06 | 0.13  ±0.04 | 0.13  ±0.04 | 0.18  ±0.07 | 0.16  ±0.06 | 0.07  ±0.02 | 0.07  ±0.02 | 0.07  ±0.02 | 0.07  ±0.05 |
|  | Cohen’s d | 0.4 | 0.04 | 0.13 | 0.23 | 0.48 | 0.24 | 0.09 | 0.15 | 0.56 | 0.14 |
| AFZ | *p*-value | 0.21 | 0.71 | 0.69 | 0.73 | **0.007** | 0.73 | 0.70 | 0.93 | **0.007** | 0.93 |
|  | trend |  |  |  |  | **↑** |  |  |  | **↓** |  |
|  | μ_pre_  ± σ_pre_ | 0.18  ±0.06 | 0.17  ±0.05 | 0.14  ±0.04 | 0.15  ±0.06 | 0.15  ±0.05 | 0.17  ±0.06 | 0.07  ±0.02 | 0.08  ±0.03 | 0.07  ±0.02 | 0.07  ±0.02 |
|  | μ_post_  ± σ_post_ | 0.17  ±0.05 | 0.18  ±0.05 | 0.13  ±0.05 | 0.13  ±0.04 | 0.18  ±0.07 | 0.16  ±0.06 | 0.07  ±0.02 | 0.08  ±0.02 | 0.06  ±0.02 | 0.07  ±0.05 |
|  | Cohen’s d | 0.23 | 0.22 | 0.08 | 0.26 | 0.52 | 0.13 | 0.10 | 0.03 | 0.48 | 0.09 |
| FZ | *p*-value | 0.66 | 0.82 | 0.74 | 0.74 | **0.02** | 0.95 | 0.96 | 0.84 | **< 0.001** | 0.84 |
|  | trend |  |  |  |  | **↑** |  |  |  | **↓** |  |
|  | μ_pre_  ± σ_pre_ | 0.18  ±0.06 | 0.17  ±0.07 | 0.13  ±0.04 | 0.15  ±0.05 | 0.15  ±0.05 | 0.17  ±0.07 | 0.08  ±0.02 | 0.08  ±0.03 | 0.07  ±0.02 | 0.07  ±0.02 |
|  | μ_post_  ± σ_post_ | 0.169  ±0.05 | 0.18  ±0.06 | 0.13  ±0.05 | 0.14  ±0.05 | 0.18  ±0.07 | 0.16  ±0.06 | 0.075  ±0.02 | 0.08  ±0.02 | 0.06  ±0.02 | 0.07  ±0.04 |
|  | Cohen’s d | 0.165 | 0.08 | 0.1 | 0.13 | 0.46 | 0.06 | 0.03 | 0.07 | 0.54 | 0.02 |
| FCZ | *p*-value | 0.66 | 0.84 | 0.89 | 0.95 | **0.007** | 0.73 | 0.73 | 0.73 | **0.02** | 0.62 |
|  | trend |  |  |  |  | **↑** |  |  |  | **↓** |  |
|  | μ_pre_  ± σ_pre_ | 0.18  ±0.06 | 0.17  ±0.06 | 0.13  ±0.04 | 0.14  ±0.05 | 0.15  ±0.06 | 0.17  ±0.07 | 0.08  ±0.02 | 0.08  ±0.02 | 0.07  ±0.02 | 0.07  ±0.02 |
|  | μ_post_  ± σ_post_ | 0.17  ±0.05 | 0.18  ±0.08 | 0.12  ±0.05 | 0.14  ±0.06 | 0.18  ±0.07 | 0.16  ±0.07 | 0.07  ±0.02 | 0.07  ±0.02 | 0.06  ±0.02 | 0.07  ±0.03 |
|  | Cohen’s d | 0.17 | 0.15 | 0.01 | 0.00 | 0.50 | 0.15 | 0.08 | 0.16 | 0.46 | 0.06 |
| CZ | *p*-value | 0.98 | 0.71 | 0.91 | 0.87 | 0.34 | 0.71 | 0.96 | 0.98 | **0.007** | 0.93 |
|  | trend |  |  |  |  |  |  |  |  | **↓** |  |
|  | μ_pre_  ± σ_pre_ | 0.18  ±0.06 | 0.18  ±0.06 | 0.12  ±0.05 | 0.14  ±0.06 | 0.16  ±0.07 | 0.19  ±0.09 | 0.07  ±0.02 | 0.07  ±0.02 | 0.08  ±0.03 | 0.07  ±0.02 |
|  | μ_post_  ± σ_post_ | 0.17  ±0.05 | 0.17  ±0.07 | 0.123  ±0.05 | 0.13  ±0.06 | 0.18  ±0.09 | 0.19  ±0.11 | 0.07  ±0.02 | 0.07  ±0.03 | 0.07  ±0.02 | 0.07  ±0.02 |
|  | Cohen’s d | 0.09 | 0.12 | 0.032 | 0.08 | 0.27 | 0.07 | 0.01 | 0.07 | 0.48 | 0.03 |
| CPZ | *p*-value | 0.51 | 0.86 | 0.52 | 0.86 | 0.43 | 0.92 | 0.96 | 0.74 | **< 0.001** | 0.73 |
|  | trend |  |  |  |  |  |  |  |  | **↓** |  |
|  | μ_pre_  ± σ_pre_ | 0.19  ±0.07 | 0.19  ±0.07 | 0.12  ±0.05 | 0.15  ±0.07 | 0.17  ±0.07 | 0.21  ±0.11 | 0.07  ±0.02 | 0.08  ±0.04 | 0.08  ±0.03 | 0.07  ±0.02 |
|  | μ_post_  ± σ_post_ | 0.17  ±0.06 | 0.18  ±0.05 | 0.13  ±0.05 | 0.14  ±0.06 | 0.18  ±0.09 | 0.21  ±0.14 | 0.07  ±0.02 | 0.08  ±0.03 | 0.07  ±0.02 | 0.07  ±0.02 |
|  | Cohen’s d | 0.18 | 0.2 | 0.14 | 0.11 | 0.22 | 0.05 | 0.04 | 0.16 | 0.60 | 0.19 |
| PZ | *p*-value | 0.20 | 0.77 | 0.83 | 0.86 | **0.02** | 0.96 | 0.83 | 0.98 | **< 0.001** | 0.28 |
|  | trend |  |  |  |  | **↑** |  |  |  | **↓** |  |
|  | μ_pre_  ± σ_pre_ | 0.19  ±0.07 | 0.18  ±0.07 | 0.13  ±0.05 | 0.15  ±0.06 | 0.16  ±0.07 | 0.19  ±0.1 | 0.07  ±0.02 | 0.09  ±0.04 | 0.08  ±0.02 | 0.07  ±0.02 |
|  | μ_post_  ± σ_post_ | 0.17  ±0.05 | 0.18  ±0.06 | 0.13  ±0.04 | 0.14  ±0.06 | 0.19  ±0.09 | 0.2  ±0.12 | 0.07  ±0.02 | 0.08  ±0.03 | 0.07  ±0.02 | 0.08  ±0.04 |
|  | Cohen’s d | 0.27 | 0.07 | 0.05 | 0.09 | 0.46 | 0.08 | 0.03 | 0.1 | 0.56 | 0.31 |
| POZ | *p*-value | 0.14 | 0.95 | 0.98 | 0.98 | **0.01** | 0.92 | 0.52 | 0.82 | **< 0.001** | 0.28 |
|  | trend |  |  |  |  | **↑** |  |  |  | **↓** |  |
|  | μ_pre_  ± σ_pre_ | 0.19  ±0.06 | 0.19  ±0.07 | 0.13  ±0.05 | 0.14  ±0.05 | 0.16  ±0.06 | 0.17  ±0.07 | 0.07  ±0.02 | 0.09  ±0.04 | 0.08  ±0.02 | 0.06  ±0.01 |
|  | μ_post_  ± σ_post_ | 0.17  ±0.05 | 0.19  ±0.06 | 0.14  ±0.06 | 0.14  ±0.06 | 0.2  ±0.08 | 0.18  ±0.09 | 0.07  ±0.02 | 0.08  ±0.03 | 0.07  ±0.02 | 0.07  ±0.05 |
|  | Cohen’s d | 0.33 | 0.02 | 0.08 | 0.02 | 0.53 | 0.16 | 0.08 | 0.24 | 0.52 | 0.28 |
| OZ | *p*-value | 0.21 | 0.93 | 0.45 | 0.73 | **0.007** | 0.92 | 0.70 | 0.87 | **0.01** | 0.85 |
|  | trend |  |  |  |  | **↑** |  |  |  | **↓** |  |
|  | μ_pre_  ± σ_pre_ | 0.19  ±0.06 | 0.18  ±0.07 | 0.13  ±0.05 | 0.14  ±0.05 | 0.16  ±0.06 | 0.17  ±0.06 | 0.07  ±0.02 | 0.09  ±0.03 | 0.08  ±0.02 | 0.07  ±0.02 |
|  | μ_post_  ± σ_post_ | 0.17  ±0.05 | 0.18  ±0.06 | 0.14  ±0.06 | 0.13  ±0.05 | 0.21  ±0.1 | 0.17  7±0.0 | 0.07  ±0.02 | 0.08  ±0.03 | 0.07  ±0.02 | 0.08  ±0.05 |
|  | Cohen’s d | 0.27 | 0.04 | 0.20 | 0.19 | 0.59 | 0.03 | 0.02 | 0.21 | 0.50 | 0.22 |
| FP2 | *p*-value | **0.03** | 0.93 | 0.59 | 0.69 | **< 0.001** | 0.73 | 0.99 | 0.92 | **0.01** | 0.93 |
|  | trend | **↓** |  |  |  | **↑** |  |  |  | **↓** |  |
|  | μ_pre_  ± σ_pre_ | 0.19  ±0.06 | 0.18  ±0.05 | 0.13  ±0.04 | 0.15  ±0.05 | 0.15  ±0.05 | 0.17  ±0.06 | 0.07  ±0.02 | 0.08  ±0.02 | 0.07  ±0.02 | 0.07  ±0.02 |
|  | μ_post_  ± σ_post_ | 0.16  ±0.05 | 0.18  ±0.05 | 0.13  ±0.05 | 0.13  ±0.04 | 0.18  ±0.07 | 0.16  ±0.06 | 0.07  ±0.02 | 0.07  ±0.02 | 0.06  ±0.01 | 0.07  ±0.05 |
|  | Cohen’s d | 0.43 | 0.06 | 0.1 | 0.25 | 0.56 | 0.14 | 0.00 | 0.13 | 0.45 | 0.07 |
| AF8 | *p*-value | 0.06 | 0.89 | 0.69 | 0.45 | **< 0.001** | 0.92 | 0.64 | 0.98 | **0.02** | 0.83 |
|  | trend |  |  |  |  | **↑** |  |  |  | **↓** |  |
|  | μ_pre_  ± σ_pre_ | 0.18  ±0.06 | 0.17  ±0.06 | 0.13  ±0.04 | 0.15  ±0.06 | 0.14  ±0.05 | 0.16  ±0.06 | 0.07  ±0.02 | 0.07  ±0.02 | 0.07  ±0.02 | 0.06  ±0.02 |
|  | μ_post_  ± σ_post_ | 0.16  ±0.05 | 0.18  ±0.06 | 0.13  ±0.05 | 0.14  ±0.05 | 0.18  ±0.07 | 0.16  ±0.070 | 0.07  ±0.03 | 0.07  ±0.02 | 0.06  ±0.01 | 0.07  ±0.04 |
|  | Cohen’s d | 0.41 | 0.11 | 0.08 | 0.24 | 0.56 | 0.04 | 0.12 | 0.02 | 0.49 | 0.16 |
| AF4 | *p*-value | **0.03** | 0.77 | 0.53 | 0.34 | **0.03** | 0.78 | 0.49 | 0.62 | 0.12 | 0.93 |
|  | trend | **↓** |  |  |  | **↑** |  |  |  |  |  |
|  | μ_pre_  ± σ_pre_ | 0.2  ±0.07 | 0.17  ±0.06 | 0.13  ±0.04 | 0.15  ±0.05 | 0.15  ±0.05 | 0.16  ±0.06 | 0.07  ±0.02 | 0.08  ±0.02 | 0.07  ±0.02 | 0.07  ±0.02 |
|  | μ_post_  ± σ_post_ | 0.17  ±0.05 | 0.18  ±0.05 | 0.13  ±0.05 | 0.13  ±0.05 | 0.18  ±0.07 | 0.16  ±0.06 | 0.07  ±0.02 | 0.07  ±0.02 | 0.06  ±0.01 | 0.07  ±0.05 |
|  | Cohen’s d | 0.43 | 0.16 | 0.11 | 0.3 | 0.48 | 0.11 | 0.13 | 0.245 | 0.37 | 0.1 |
| F8 | *p*-value | 0.21 | 0.92 | 0.59 | 0.51 | **< 0.001** | 0.98 | 0.78 | 0.93 | 0.71 | 0.84 |
|  | trend |  |  |  |  | **↓** |  |  |  |  |  |
|  | μ_pre_  ± σ_pre_ | 0.18  ±0.06 | 0.18  ±0.06 | 0.13  ±0.05 | 0.15  ±0.06 | 0.14  ±0.05 | 0.16  ±0.06 | 0.07  ±0.02 | 0.07  ±0.03 | 0.07  ±0.02 | 0.07  ±0.02 |
|  | μ_post_  ± σ_post_ | 0.17  ±0.05 | 0.18  ±0.07 | 0.12  ±0.05 | 0.14  ±0.05 | 0.17  ±0.07 | 0.17  ±0.07 | 0.07  ±0.02 | 0.07  ±0.02 | 0.07  ±0.02 | 0.07  ±0.05 |
|  | Cohen’s d | 0.30 | 0.02 | 0.11 | 0.27 | 0.5 | 0.04 | 0.05 | 0.08 | 0.12 | 0.13 |
| F6 | *p*-value | 0.20 | 0.82 | 0.83 | 0.36 | **0.05** | 0.82 | 0.21 | 0.47 | 0.17 | 0.93 |
|  | trend |  |  |  |  | **↑** |  |  |  |  |  |
|  | μ_pre_  ± σ_pre_ | 0.18  ±0.06 | 0.18  ±0.07 | 0.13  ±0.05 | 0.15  ±0.06 | 0.14  ±0.05 | 0.16  ±0.06 | 0.07  ±0.02 | 0.08  ±0.03 | 0.08  ±0.02 | 0.07  ±0.02 |
|  | μ_post_  ± σ_post_ | 0.17  ±0.05 | 0.19  ±0.07 | 0.13  ±0.05 | 0.13  ±0.06 | 0.17  ±0.07 | 0.160  ±0.07 | 0.08  ±0.03 | 0.07  ±0.02 | 0.07  ±0.02 | 0.07  ±0.05 |
|  | Cohen’s d | 0.29 | 0.14 | 0.02 | 0.29 | 0.45 | 0.05 | 0.26 | 0.26 | 0.33 | 0.13 |
| F4 | *p*-value | 0.70 | 0.84 | 1.00 | 0.47 | 0.21 | 0.95 | 0.21 | 1.00 | **0.02** | 0.93 |
|  | trend |  |  |  |  |  |  |  |  | **↓** |  |
|  | μ_pre_  ± σ_pre_ | 0.18  ±0.06 | 0.175  ±0.061 | 0.13  ±0.05 | 0.15  ±0.05 | 0.15  ±0.05 | 0.16  ±0.06 | 0.07  ±0.02 | 0.08  ±0.03 | 0.08  ±0.02 | 0.07  ±0.02 |
|  | μ_post_  ± σ_post_ | 0.17  ±0.05 | 0.18  ±0.06 | 0.13  ±0.05 | 0.14  ±0.06 | 0.17  ±0.07 | 0.16  ±0.06 | 0.08  ±0.02 | 0.08  ±0.02 | 0.07  ±0.02 | 0.07  ±0.04 |
|  | Cohen’s d | 0.09 | 0.12 | 0.01 | 0.17 | 0.36 | 0.02 | 0.26 | 0.04 | 0.43 | 0.15 |
| F2 | *p*-value | 0.32 | 0.92 | 0.61 | 0.49 | **0.05** | 0.95 | 0.64 | 0.94 | **0.007** | 0.98 |
|  | trend |  |  |  |  | **↑** |  |  |  | **↓** |  |
|  | μ_pre_  ± σ_pre_ | 0.19  ±0.06 | 0.17  ±0.06 | 0.13  ±0.04 | 0.15  ±0.05 | 0.16  ±0.05 | 0.16  ±0.06 | 0.07  ±0.02 | 0.08  ±0.02 | 0.07  ±0.02 | 0.07  ±0.02 |
|  | μ_post_  ± σ_post_ | 0.17  ±0.05 | 0.18  ±0.07 | 0.13  ±0.05 | 0.14  ±0.06 | 0.18  ±0.07 | 0.17  ±0.06 | 0.08  ±0.02 | 0.08  ±0.02 | 0.07  ±0.02 | 0.07  ±0.04 |
|  | Cohen’s d | 0.24 | 0.11 | 0.17 | 0.15 | 0.43 | 0.01 | 0.12 | 0.02 | 0.50 | 0.13 |
| FT8 | *p*-value | 0.22 | 0.94 | 0.65 | 0.77 | 0.06 | 0.92 | 0.12 | 1.00 | **< 0.001** | 0.77 |
|  | trend |  |  |  |  |  |  |  |  | **↓** |  |
|  | μ_pre_  ± σ_pre_ | 0.19  ±0.06 | 0.18  ±0.07 | 0.12  ±0.05 | 0.15  ±0.07 | 0.14  ±0.06 | 0.16  ±0.07 | 0.07  ±0.02 | 0.07  ±0.03 | 0.09  ±0.04 | 0.07  ±0.02 |
|  | μ_post_  ± σ_post_ | 0.17  ±0.05 | 0.17  ±0.07 | 0.12  ±0.04 | 0.14  ±0.05 | 0.16  ±0.07 | 0.16  ±0.07 | 0.07  ±0.03 | 0.07  ±0.02 | 0.07  ±0.02 | 0.07  ±0.06 |
|  | Cohen’s d | 0.29 | 0.04 | 0.13 | 0.23 | 0.34 | 0.04 | 0.18 | 0.11 | 0.67 | 0.21 |
| FC6 | *p*-value | 0.59 | 0.84 | 0.72 | 0.20 | 0.27 | 0.73 | 0.64 | 0.93 | 0.93 | 0.92 |
|  | trend |  |  |  |  |  |  |  |  |  |  |
|  | μ_pre_  ± σ_pre_ | 0.18  ±0.06 | 0.19  ±0.08 | 0.13  ±0.05 | 0.15  ±0.06 | 0.14  ±0.05 | 0.16  ±0.06 | 0.07  ±0.02 | 0.08  ±0.02 | 0.08  ±0.02 | 0.07  ±0.02 |
|  | μ_post_  ± σ_post_ | 0.17  ±0.05 | 0.2  ±0.08 | 0.13  ±0.06 | 0.13  ±0.05 | 0.15  ±0.07 | 0.15  ±0.05 | 0.07  ±0.03 | 0.08  ±0.02 | 0.07  ±0.02 | 0.07  ±0.05 |
|  | Cohen’s d | 0.13 | 0.11 | 0.01 | 0.42 | 0.31 | 0.2 | 0.17 | 0.08 | 0.07 | 0.16 |
| FC4 | *p*-value | 0.30 | 0.28 | 0.82 | 0.82 | 0.42 | 0.86 | 0.96 | 0.71 | 0.40 | 0.87 |
|  | trend |  |  |  |  |  |  |  |  |  |  |
|  | μ_pre_  ± σ_pre_ | 0.19  ±0.07 | 0.18  ±0.07 | 0.13  ±0.06 | 0.13  ±0.05 | 0.15  ±0.06 | 0.15  ±0.05 | 0.07  ±0.02 | 0.08  ±0.03 | 0.08  ±0.03 | 0.07  ±0.02 |
|  | μ_post_  ± σ_post_ | 0.18  ±0.06 | 0.20  ±0.08 | 0.14  ±0.07 | 0.12  ±0.05 | 0.16  ±0.08 | 0.15  ±0.06 | 0.08  ±0.03 | 0.08  ±0.03 | 0.07  ±0.02 | 0.07  ±0.04 |
|  | Cohen’s d | 0.21 | 0.33 | 0.15 | 0.14 | 0.27 | 0.10 | 0.08 | 0.11 | 0.2 | 0.14 |
| FC2 | *p*-value | 0.73 | 0.82 | 0.97 | 0.40 | 0.28 | 1.00 | 0.83 | 0.71 | **< 0.001** | 0.93 |
|  | trend |  |  |  |  |  |  |  |  | **↓** |  |
|  | μ_pre_  ± σ_pre_ | 0.18  ±0.06 | 0.17  ±0.06 | 0.13  ±0.04 | 0.14  ±0.05 | 0.15  ±0.05 | 0.15  ±0.05 | 0.08  ±0.02 | 0.07  ±0.02 | 0.08  ±0.02 | 0.07  ±0.02 |
|  | μ_post_  ± σ_post_ | 0.18  ±0.06 | 0.19  ±0.08 | 0.13  ±0.06 | 0.13  ±0.05 | 0.172  ±0.07 | 0.16  ±0.07 | 0.08  ±0.02 | 0.08  ±0.02 | 0.07  ±0.02 | 0.07  ±0.04 |
|  | Cohen’s d | 0.08 | 0.18 | 0.14 | 0.22 | 0.29 | 0.13 | 0.03 | 0.17 | 0.43 | 0.09 |
| T4 | *p*-value | 0.67 | 0.89 | 0.21 | 0.92 | 0.67 | 0.87 | 0.47 | 0.93 | 0.20 | 0.92 |
|  | trend |  |  |  |  |  |  |  |  |  |  |
|  | μ_pre_  ± σ_pre_ | 0.18  ±0.06 | 0.19  ±0.07 | 0.13  ±0.05 | 0.14  ±0.06 | 0.15  ±0.06 | 0.17  ±0.07 | 0.08  ±0.02 | 0.07  ±0.03 | 0.07  ±0.03 | 0.07  ±0.02 |
|  | μ_post_  ± σ_post_ | 0.17  ±0.06 | 0.18  ±0.06 | 0.11  ±0.04 | 0.14  ±0.05 | 0.16  ±0.08 | 0.17  ±0.08 | 0.07  ±0.03 | 0.07  ±0.02 | 0.08  ±0.02 | 0.08  ±0.05 |
|  | Cohen’s d | 0.11 | 0.09 | 0.3 | 0.01 | 0.21 | 0.02 | 0.10 | 0.03 | 0.16 | 0.14 |
| C6 | *p*-value | 0.89 | 0.84 | 0.64 | 0.47 | 0.61 | 0.36 | 0.34 | 0.77 | 0.62 | 1.00 |
|  | trend |  |  |  |  |  |  |  |  |  |  |
|  | μ_pre_  ± σ_pre_ | 0.18  ±0.07 | 0.19  ±0.07 | 0.14  ±0.06 | 0.14  ±0.05 | 0.14  ±0.05 | 0.16  ±0.07 | 0.08  ±0.02 | 0.08  ±0.03 | 0.08  ±0.03 | 0.07  ±0.02 |
|  | μ_post_  ± σ_post_ | 0.18  ±0.06 | 0.2  ±0.07 | 0.13  ±0.06 | 0.13  ±0.05 | 0.15  ±0.08 | 0.15  ±0.06 | 0.07  ±0.02 | 0.08  ±0.02 | 0.07  ±0.02 | 0.07  ±0.06 |
|  | Cohen’s d | 0.08 | 0.11 | 0.09 | 0.28 | 0.27 | 0.26 | 0.15 | 0.04 | 0.29 | 0.16 |
| C4 | *p*-value | 0.65 | 0.73 | 0.82 | 0.32 | 0.58 | 0.71 | 0.75 | 0.87 | 0.40 | 0.92 |
|  | trend |  |  |  |  |  |  |  |  |  |  |
|  | μ_pre_  ± σ_pre_ | 0.19  ±0.07 | 0.19  ±0.07 | 0.13  ±0.06 | 0.13  ±0.05 | 0.15  ±0.04 | 0.16  ±0.08 | 0.08  ±0.02 | 0.08  ±0.03 | 0.08  ±0.03 | 0.07  ±0.02 |
|  | μ_post_  ± σ_post_ | 0.18  ±0.07 | 0.20  ±0.08 | 0.14  ±0.06 | 0.12  ±0.05 | 0.17  ±0.10 | 0.14  ±0.05 | 0.08  ±0.03 | 0.08  ±0.03 | 0.07  ±0.02 | 0.07  ±0.04 |
|  | Cohen’s d | 0.13 | 0.20 | 0.1 | 0.26 | 0.31 | 0.27 | 0.01 | 0.08 | 0.26 | 0.18 |
| C2 | *p*-value | 0.57 | 0.73 | 0.61 | 0.28 | 0.83 | 0.92 | 0.57 | 0.93 | 0.06 | 0.92 |
|  | trend |  |  |  |  |  |  |  |  |  |  |
|  | μ_pre_  ± σ_pre_ | 0.19  ±0.07 | 0.17  ±0.06 | 0.12  ±0.04 | 0.14  ±0.05 | 0.15  ±0.06 | 0.16  ±0.07 | 0.07  ±0.03 | 0.07  ±0.02 | 0.08  ±0.03 | 0.07  ±0.02 |
|  | μ_post_  ± σ_post_ | 0.18  ±0.06 | 0.19  ±0.08 | 0.13  ±0.05 | 0.13  ±0.05 | 0.17  ±0.1 | 0.17  ±0.09 | 0.08  ±0.02 | 0.08  ±0.03 | 0.07  ±0.03 | 0.07  ±0.03 |
|  | Cohen’s d | 0.13 | 0.21 | 0.20 | 0.29 | 0.24 | 0.15 | 0.07 | 0.16 | 0.37 | 0.11 |
| TP8 | *p*-value | 0.82 | 0.93 | 0.43 | 0.87 | 0.65 | 0.93 | 0.43 | 0.98 | 0.62 | 0.48 |
|  | trend |  |  |  |  |  |  |  |  |  |  |
|  | μ_pre_  ± σ_pre_ | 0.18  ±0.06 | 0.18  ±0.07 | 0.13  ±0.05 | 0.14  ±0.05 | 0.16  ±0.06 | 0.18  ±0.08 | 0.07  ±0.02 | 0.08  ±0.03 | 0.08  ±0.03 | 0.07  ±0.01 |
|  | μ_post_  ± σ_post_ | 0.17  ±0.06 | 0.184  ±0.06 | 0.12  ±0.05 | 0.14  ±0.06 | 0.18  ±0.10 | 0.18  ±0.1 | 0.07  ±0.02 | 0.08  ±0.03 | 0.07  ±0.02 | 0.08  ±0.06 |
|  | Cohen’s d | 0.07 | 0.01 | 0.14 | 0.08 | 0.27 | 0.05 | 0.15 | 0.01 | 0.28 | 0.26 |
| CP6 | *p*-value | 0.64 | 0.95 | 0.52 | 0.84 | 0.23 | 0.92 | 0.52 | 0.87 | **0.02** | 0.89 |
|  | trend |  |  |  |  |  |  |  |  | **↓** |  |
|  | μ_pre_  ± σ_pre_ | 0.18  ±0.07 | 0.18  ±0.07 | 0.14  ±0.05 | 0.14  ±0.05 | 0.15  ±0.05 | 0.16  ±0.07 | 0.08  ±0.02 | 0.08  ±0.03 | 0.08  ±0.04 | 0.07  ±0.02 |
|  | μ_post_  ± σ_post_ | 0.18  ±0.05 | 0.18  ±0.05 | 0.13  ±0.05 | 0.14  ±0.05 | 0.17  ±0.09 | 0.17  ±0.08 | 0.08  ±0.02 | 0.08  ±0.03 | 0.07  ±0.02 | 0.08  ±0.05 |
|  | Cohen’s d | 0.19 | 0.11 | 0.15 | 0.12 | 0.4 | 0.08 | 0.13 | 0.03 | 0.49 | 0.19 |
| CP4 | *p*-value | 0.59 | 0.94 | 0.87 | 0.32 | 0.37 | 0.73 | 0.67 | 0.84 | **0.04** | 0.92 |
|  | trend |  |  |  |  |  |  |  |  | **↓** |  |
|  | μ_pre_  ± σ_pre_ | 0.19  ±0.07 | 0.19  ±0.06 | 0.14  ±0.05 | 0.15  ±0.06 | 0.15  ±0.06 | 0.18  ±0.11 | 0.08  ±0.02 | 0.07  ±0.03 | 0.08  ±0.03 | 0.07  ±0.01 |
|  | μ_post_  ± σ_post_ | 0.18  ±0.06 | 0.19  ±0.06 | 0.13  ±0.05 | 0.13  ±0.05 | 0.18  ±0.11 | 0.15  ±0.06 | 0.08  ±0.02 | 0.08  ±0.03 | 0.07  ±0.02 | 0.07  ±0.05 |
|  | Cohen’s d | 0.17 | 0.02 | 0.05 | 0.31 | 0.41 | 0.33 | 0.03 | 0.12 | 0.44 | 0.15 |
| CP2 | *p*-value | 0.83 | 0.73 | 0.47 | 0.51 | 0.21 | 0.73 | 0.83 | 0.92 | **< 0.001** | 0.98 |
|  | trend |  |  |  |  |  |  |  |  | **↓** |  |
|  | μ_pre_  ± σ_pre_ | 0.19  ±0.07 | 0.18  ±0.06 | 0.13  ±0.05 | 0.15  ±0.06 | 0.15  ±0.06 | 0.18  ±0.09 | 0.07  ±0.02 | 0.08  ±0.03 | 0.08  ±0.03 | 0.07  ±0.02 |
|  | μ_post_  ± σ_post_ | 0.18  ±0.06 | 0.18  ±0.06 | 0.13  ±0.05 | 0.13  ±0.06 | 0.18  ±0.10 | 0.20  ±0.11 | 0.07  ±0.02 | 0.08  ±0.03 | 0.07  ±0.02 | 0.07  ±0.03 |
|  | Cohen’s d | 0.09 | 0.14 | 0.15 | 0.26 | 0.39 | 0.24 | 0.04 | 0.00 | 0.48 | 0.12 |
| T6 | *p*-value | 0.06 | 0.80 | 0.45 | 0.73 | 0.22 | 0.95 | 0.86 | 0.99 | 0.41 | 0.28 |
|  | trend |  |  |  |  |  |  |  |  |  |  |
|  | μ_pre_  ± σ_pre_ | 0.18  ±0.06 | 0.19  ±0.06 | 0.13  ±0.05 | 0.14  ±0.05 | 0.16  ±0.07 | 0.18  ±0.09 | 0.07  ±0.02 | 0.08  ±0.03 | 0.07  ±0.03 | 0.07  ±0.02 |
|  | μ_post_  ± σ_post_ | 0.16  ±0.05 | 0.18  ±0.06 | 0.13  ±0.05 | 0.14  ±0.06 | 0.2  ±0.11 | 0.18  ±0.1 | 0.07  ±0.02 | 0.08  ±0.02 | 0.07  ±0.02 | 0.08  ±0.06 |
|  | Cohen’s d | 0.37 | 0.11 | 0.11 | 0.12 | 0.38 | 0.02 | 0.00 | 0.12 | 0.32 | 0.30 |
| P6 | *p*-value | 0.21 | 0.85 | 0.59 | 0.93 | **0.02** | 0.87 | 0.64 | 0.92 | **< 0.001** | 0.95 |
|  | trend |  |  |  |  | **↑** |  |  |  | **↓** |  |
|  | μ_pre_  ± σ_pre_ | 0.18  ±0.06 | 0.18  ±0.06 | 0.13  ±0.05 | 0.14  ±0.05 | 0.15  ±0.05 | 0.13  ±0.09 | 0.08  ±0.02 | 0.08  ±0.03 | 0.08  ±0.03 | 0.07  ±0.02 |
|  | μ_post_  ± σ_post_ | 0.17  ±0.05 | 0.176  ±0.05 | 0.13  ±0.05 | 0.14  ±0.05 | 0.19  ±0.09 | 0.18  ±0.09 | 0.07  ±0.02 | 0.08  ±0.03 | 0.067  ±0.02 | 0.07  ±0.05 |
|  | Cohen’s d | 0.29 | 0.14 | 0.09 | 0.01 | 0.52 | 0.09 | 0.12 | 0.01 | 0.6 | 0.14 |
| P4 | *p*-value | 0.30 | 0.94 | 0.50 | 0.71 | **0.01** | 0.95 | 0.49 | 0.93 | **0.04** | 0.95 |
|  | trend |  |  |  |  | **↑** |  |  |  | **↓** |  |
|  | μ_pre_  ± σ_pre_ | 0.18  ±0.07 | 0.18  ±0.06 | 0.13  ±0.04 | 0.14  ±0.05 | 0.14  ±0.05 | 0.18  ±0.08 | 0.08  ±0.02 | 0.08  ±0.026 | 0.08  ±0.03 | 0.07  ±0.02 |
|  | μ_post_  ± σ_post_ | 0.17  ±0.05 | 0.18  ±0.05 | 0.13  ±0.05 | 0.13  ±0.05 | 0.18  ±0.09 | 0.17  ±0.07 | 0.07  ±0.02 | 0.08  ±0.03 | 0.07  ±0.02 | 0.07  ±0.05 |
|  | Cohen’s d | 0.23 | 0.07 | 0.08 | 0.16 | 0.54 | 0.12 | 0.15 | 0.02 | 0.48 | 0.15 |
| P2 | *p*-value | 0.78 | 0.92 | 0.87 | 0.84 | **0.02** | 0.92 | 0.96 | 0.90 | **0.007** | 0.84 |
|  | trend |  |  |  |  | **↑** |  |  |  | **↓** |  |
|  | μ_pre_  ± σ_pre_ | 0.18  ±0.07 | 0.18  ±0.06 | 0.13  ±0.04 | 0.14  ±0.06 | 0.14  ±0.06 | 0.19  ±0.1 | 0.07  ±0.02 | 0.08  ±0.03 | 0.08  ±0.03 | 0.07  ±0.02 |
|  | μ_post_  ± σ_post_ | 0.17  ±0.05 | 0.18  ±0.05 | 0.13  ±0.05 | 0.14  ±0.06 | 0.18  ±0.08 | 0.19  ±0.09 | 0.07  ±0.02 | 0.08  ±0.03 | 0.07  ±0.02 | 0.08  ±0.05 |
|  | Cohen’s d | 0.12 | 0.01 | 0.03 | 0.12 | 0.47 | 0.05 | 0.02 | 0.01 | 0.55 | 0.20 |
| PO8 | *p*-value | 0.10 | 0.84 | 0.83 | 0.78 | **0.03** | 0.86 | 0.42 | 0.93 | 0.42 | 0.87 |
|  | trend |  |  |  |  | **↑** |  |  |  |  |  |
|  | μ_pre_  ± σ_pre_ | 0.18  ±0.06 | 0.19  ±0.06 | 0.13  ±0.05 | 0.14  ±0.05 | 0.16  ±0.07 | 0.19  ±0.09 | 0.08  ±0.02 | 0.08  ±0.03 | 0.07  ±0.03 | 0.07  ±0.02 |
|  | μ_post_  ± σ_post_ | 0.16  ±0.05 | 0.18  ±0.06 | 0.13  ±0.05 | 0.14  ±0.05 | 0.20  ±0.10 | 0.18  ±0.1 | 0.07  ±0.02 | 0.08  ±0.02 | 0.07  ±0.02 | 0.08  ±0.05 |
|  | Cohen’s d | 0.41 | 0.12 | 0.03 | 0.11 | 0.47 | 0.08 | 0.15 | 0.07 | 0.28 | 0.2 |
| PO4 | *p*-value | 0.43 | 0.92 | 0.99 | 0.93 | **< 0.001** | 0.87 | 0.69 | 0.92 | 0.08 | 0.82 |
|  | trend |  |  |  |  | **↑** |  |  |  |  |  |
|  | μ_pre_  ± σ_pre_ | 0.18  ±0.07 | 0.18  ±0.06 | 0.13  ±0.05 | 0.13  ±0.05 | 0.15  ±0.06 | 0.18  ±0.1 | 0.07  ±0.02 | 0.08  ±0.03 | 0.08  ±0.03 | 0.07  ±0.02 |
|  | μ_post_  ± σ_post_ | 0.17  ±0.05 | 0.18  ±0.05 | 0.13  ±0.06 | 0.14  ±0.06 | 0.19  ±0.08 | 0.16  ±0.07 | 0.07  ±0.02 | 0.08  ±0.03 | 0.07  ±0.02 | 0.08  ±0.05 |
|  | Cohen’s d | 0.20 | 0.01 | 0.06 | 0.08 | 0.61 | 0.18 | 0.04 | 0.11 | 0.40 | 0.22 |
| O2 | *p*-value | 0.17 | 0.94 | 0.59 | 0.73 | **0.02** | 0.84 | 0.43 | 0.92 | **0.04** | 0.98 |
|  | trend |  |  |  |  | **↑** |  |  |  | **↓** |  |
|  | μ_pre_  ± σ_pre_ | 0.18  ±0.06 | 0.19  ±0.07 | 0.13  ±0.05 | 0.14  ±0.05 | 0.16  ±0.06 | 0.17  ±0.07 | 0.08  ±0.02 | 0.08  ±0.03 | 0.08  ±0.02 | 0.07  ±0.02 |
|  | μ_post_  ± σ_post_ | 0.16  ±0.05 | 0.18  ±0.06 | 0.14  ±0.06 | 0.14  ±0.05 | 0.20  ±0.10 | 0.17  ±0.08 | 0.07  ±0.02 | 0.08  ±0.02 | 0.07  ±0.02 | 0.08  ±0.05 |
|  | Cohen’s d | 0.29 | 0.09 | 0.18 | 0.16 | 0.49 | 0.09 | 0.15 | 0.01 | 0.40 | 0.18 |

**Supplementary Table 2:** Nonstatistically significant results of the u test of PLI for single scalp regions analyses. Values are reported as p-value, μ_pre_ ± σ_pre_ (i.e., mean ± standard deviation before VNS), μ_post_ ± σ_post_ (i.e., mean ± standard deviation after VNS), and Cohen’s d (i.e., effect size).

|  |  | *Global* | | *Frontal* | | *Temporal* | | *Occipital* | | *Parietal* | | *Central* | |
| --- | --- | --- | --- | --- | --- | --- | --- | --- | --- | --- | --- | --- | --- |
|  |  | *R* | *NR* | *R* | *NR* | *R* | *NR* | *R* | *NR* | *R* | *NR* | *R* | *NR* |
| Theta | *p*-value | 0.68 | 0.18 | 0.68 | 0.18 | 0.98 | 0.41 | 0.36 | 0.33 | 0.53 | 0.69 | 0.75 | 0.41 |
|  | trend |  |  |  |  |  |  |  |  |  |  |  |  |
|  | μ_pre_  ± σ_pre_ | 0.13  ±0.03 | 0.14  ±0.04 | 0.13  ±0.03 | 0.14  ±0.04 | 0.13  ±0.05 | 0.15  ±0.05 | 0.12  ±0.05 | 0.15  ±0.06 | 0.13  ±0.05 | 0.14  ±0.05 | 0.13  ±0.04 | 0.15  ±0.05 |
|  | μ_post_  ± σ_post_ | 0.13  ±0.03 | 0.13  ±0.04 | 0.13  ±0.03 | 0.13  ±0.04 | 0.13  ±0.05 | 0.14  ±0.05 | 0.12  ±0.04 | 0.13  ±0.05 | 0.13  ±0.05 | 0.14  ±0.06 | 0.13  ±0.04 | 0.14  ±0.05 |
|  | Cohen’s d | 0.03 | 0.28 | 0.03 | 0.28 | 0.01 | 0.23 | 0.20 | 0.36 | 0.11 | 0.14 | 0.03 | 0.17 |
| Beta | *p*-value | 0.56 | 0.69 | 0.56 | 0.69 | 0.53 | 0.90 | 0.58 | 0.41 | 0.13 | 0.69 | 0.45 | 0.90 |
|  | trend |  |  |  |  |  |  |  |  |  |  |  |  |
|  | μ_pre_  ± σ_pre_ | 0.07  ±0.01 | 0.08  ±0.02 | 0.07  ±0.01 | 0.079  ±0.02 | 0.07  ±0.02 | 0.08  ±0.02 | 0.07  ±0.02 | 0.07  ±0.02 | 0.08  ±0.03 | 0.09  ±0.05 | 0.08  ±0.02 | 0.08  ±0.03 |
|  | μ_post_  ± σ_post_ | 0.07  ±0.01 | 0.08  ±0.01 | 0.07  ±0.01 | 0.08  ±0.01 | 0.08  ±0.02 | 0.07  ±0.02 | 0.07  ±0.02 | 0.08  ±0.02 | 0.07  ±0.03 | 0.08  ±0.03 | 0.07  ±0.02 | 0.08  ±0.02 |
|  | Cohen’s d | 0.03 | 0.20 | 0.03 | 0.20 | 0.10 | 0.05 | 0.09 | 0.11 | 0.30 | 0.23 | 0.19 | 0.09 |

**Supplementary Table 3**: Results of the u test of aperiodic parameters for single-channel analyses. Values are reported as p-value, μ_pre_ ± σ_pre_ (i.e., mean ± standard deviation before VNS), μ_post_ ± σ_post_ (i.e., mean ± standard deviation after VNS), and Cohen’s d (i.e., effect size). R = responders; NR = non-responders. Statistically significant results are highlighted in bold. The direction of arrows represents the trend of change: upward stands for a statistically significant increase and downward for a decrease.

|  |  | | *Offset* | | | | *Exponent* | | | | |
| --- | --- | --- | --- | --- | --- | --- | --- | --- | --- | --- | --- |
|  |  | | *R* | | *NR* | | *R* | | | *NR* | |
| FP1 | *p*-value | | 0.17 | | **<0.001** | | 0.99 | | | **<0.001** | |
|  | trend | |  | | **↑** | |  | | | **↑** | |
|  | μ_pre_ | | 1.92 | | 2.20 | | 2.09 | | | 2.29 | |
|  | ± σ_pre_ | | ±0.72 | | ±0.4 | | ±0.35 | | | ±0.45 | |
|  | μ_post_ | | 1.78 | | 2.46 | | 2.1 | | | 2.56 | |
|  | ± σ_post_ | | ±0.38 | | ±0.6 | | ±0.32 | | | ±0.44 | |
|  | Cohen’s d | | 0.23 | | 0.51 | | 0.03 | | | 0.6 | |
| AF7 | *p*-value | | 0.19 | | **<0.001** | | 0.46 | | | **<0.001** | |
|  | trend | |  | | **↑** | |  | | | **↑** | |
|  | μ_pre_ | | 1.9 | | 2.18 | | 2.03 | | | 2.23 | |
|  | ± σ_pre_ | | ±0.45 | | ±0.43 | | ±0.30 | | | ±0.46 | |
|  | μ_post_ | | 1.83 | | 2.41 | | 2.09 | | | 2.46 | |
|  | ± σ_post_ | | ±0.38 | | ±0.56 | | ±0.31 | | | ±0.46 | |
|  | Cohen’s d | | 0.16 | | 0.47 | | 0.20 | | | 0.49 | |
| AF3 | *p*-value | | 0.10 | | **0.007** | | 0.94 | | | **0.008** | |
|  | trend | |  | | **↑** | |  | | | **↑** | |
|  | μ_pre_ | | 1.75 | | 2.11 | | 2.14 | | | 2.34 | |
|  | ± σ_pre_ | | ±0.48 | | ±0.42 | | ±0.26 | | | ±0.44 | |
|  | μ_post_ | | 1.65 | | 2.33 | | 2.13 | | | 2.54 | |
|  | ± σ_post_ | | ±0.43 | | ±0.6 | | ±0.30 | | | ±0.46 | |
|  | Cohen’s d | | 0.23 | | 0.43 | | 0.02 | | | 0.46 | |
| F7 | *p*-value | | **0.006** | | **0.03** | | **<0.001** | | | **0.009** | |
|  | trend | | **↓** | | **↑** | | **↓** | | | **↑** | |
|  | μ_pre_ | | 1.92 | | 2.15 | | 1.98 | | | 2.19 | |
|  | ± σ_pre_ | | ±0.41 | | ±0.37 | | ±0.34 | | | ±0.5 | |
|  | μ_post_ | | 1.73 | | 2.31 | | 1.75 | | | 2.4 | |
|  | ± σ_post_ | | ±0.42 | | ±0.52 | | ±0.37 | | | ±0.5 | |
|  | Cohen’s d | | 0.48 | | 0.34 | | 0.65 | | | 0.43 | |
|  | |  | | *Offset* | | | | *Exponent* | | | |
|  | |  | | *R* | | *NR* | | *R* | *NR* | | |
| F5 | | *p*-value | | 0.13 | | **0.003** | | 0.46 | **0.02** | | |
|  |  | trend | |  | | **↑** | |  | **↑** | | |
|  |  | μ_pre_ | | 1.74 | | 1.98 | | 1.98 | 2.2 | | |
|  |  | ± σ_pre_ | | ±0.46 | | ±0.47 | | ±0.33 | ±0.52 | | |
|  |  | μ_post_ | | 1.64 | | 2.22 | | 1.95 | 2.38 | | |
|  |  | ± σ_post_ | | ±0.42 | | ±0.61 | | ±0.3 | ±0.54 | | |
|  |  | Cohen’s d | | 0.24 | | 0.44 | | 0.11 | 0.36 | | |
| F3 | | *p*-value | | 0.18 | | **<0.001** | | 0.94 | **<0.001** | | |
|  |  | trend | |  | | **↑** | |  | **↑** | | |
|  |  | μ_pre_ | | 1.63 | | 1.92 | | 2.05 | 2.25 | | |
|  |  | ± σ_pre_ | | ±0.48 | | ±0.51 | | ±0.29 | ±0.52 | | |
|  |  | μ_post_ | | 1.56 | | 2.23 | | 2.05 | 2.51 | | |
|  |  | ± σ_post_ | | ±0.39 | | ±0.65 | | ±0.29 | ±0.46 | | |
|  |  | Cohen’s d | | 0.17 | | 0.53 | | 0.01 | 0.53 | | |
| F1 | | *p*-value | | 0.45 | | **0.02** | | 0.29 | **0.008** | | |
|  |  | trend | |  | | **↑** | |  | **↑** | | |
|  |  | μ_pre_ | | 1.58 | | 1.98 | | 2.10 | 2.34 | | |
|  |  | ± σ_pre_ | | ±0.49 | | ±0.47 | | ±0.23 | ±0.44 | | |
|  |  | μ_post_ | | 1.53 | | 2.17 | | 2.14 | 2.54 | | |
|  |  | ± σ_post_ | | ±0.42 | | ±0.65 | | ±0.30 | ±0.47 | | |
|  |  | Cohen’s d | | 0.1 | | 0.33 | | 0.12 | 0.44 | | |
| FT7 | | *p*-value | | **0.03** | | **0.007** | | **0.009** | **0.003** | | |
|  |  | trend | | **↓** | | **↑** | | **↓** | **↑** | | |
|  |  | μ_pre_ | | 1.83 | | 2.04 | | 1.92 | 2.14 | | |
|  |  | ± σ_pre_ | | ±0.39 | | ±0.38 | | ±0.35 | ±0.52 | | |
|  |  | μ_post_ | | 1.67 | | 2.24 | | 1.77 | 2.40 | | |
|  |  | ± σ_post_ | | ±0.44 | | ±0.45 | | ±0.38 | ±0.48 | | |
|  |  | Cohen’s d | | 0.39 | | 0.46 | | 0.4 | 0.53 | | |
|  | |  | | *Offset* | | | | *Exponent* | | | |
|  | |  | | *R* | | *NR* | | *R* | | | *NR* |
| FC5 | | *p*-value | | **0.06** | | **0.007** | | **0.05** | | | **0.006** |
|  |  | trend | | **↓** | | **↑** | | **↓** | | | **↑** |
|  |  | μ_pre_ | | 1.65 | | 1.92 | | 1.92 | | | 2.2 |
|  |  | ± σ_pre_ | | ±0.42 | | ±0.45 | | ±0.32 | | | ±0.52 |
|  |  | μ_post_ | | 1.50 | | 2.15 | | 1.79 | | | 2.42 |
|  |  | ± σ_post_ | | ±0.45 | | ±0.57 | | ±0.42 | | | ±0.53 |
|  |  | Cohen’s d | | 0.34 | | 0.44 | | 0.36 | | | 0.44 |
| FC3 | | *p*-value | | 0.23 | | **0.007** | | 1.00 | | | **0.006** |
|  |  | trend | |  | | **↑** | |  | | | **↑** |
|  |  | μ_pre_ | | 1.43 | | 1.79 | | 1.96 | | | 2.28 |
|  |  | ± σ_pre_ | | ±0.49 | | ±0.51 | | ±0.34 | | | ±0.47 |
|  |  | μ_post_ | | 1.34 | | 2.03 | | 1.98 | | | 2.50 |
|  |  | ± σ_post_ | | ±0.42 | | ±0.63 | | ±0.32 | | | ±0.5 |
|  |  | Cohen’s d | | 0.19 | | 0.42 | | 0.04 | | | 0.46 |
| FC1 | | *p*-value | | 0.11 | | **<0.001** | | 0.44 | | | **0.003** |
|  |  | trend | |  | | **↑** | |  | | | **↑** |
|  |  | μ_pre_ | | 1.45 | | 1.69 | | 2.09 | | | 2.28 |
|  |  | ± σ_pre_ | | ±0.48 | | ±0.57 | | ±0.24 | | | ±0.49 |
|  |  | μ_post_ | | 1.35 | | 2.02 | | 2.10 | | | 2.53 |
|  |  | ± σ_post_ | | ±0.43 | | ±0.68 | | ±0.29 | | | ±0.47 |
|  |  | Cohen’s d | | 0.22 | | 0.54 | | 0.05 | | | 0.52 |
| T3 | | *p*-value | | **0.006** | | **<0.001** | | **<0.001** | | | 0.08 |
|  |  | trend | | **↓** | | **↑** | | **↓** | | |  |
|  |  | μ_pre_ | | 1.73 | | 1.84 | | 1.87 | | | 2.14 |
|  |  | ± σ_pre_ | | ±0.39 | | ±0.49 | | ±0.35 | | | ±0.52 |
|  |  | μ_post_ | | 1.52 | | 2.13 | | 1.52 | | | 2.3 |
|  |  | ± σ_post_ | | ±0.45 | | ±0.55 | | ±0.30 | | | ±0.60 |
|  |  | Cohen’s d | | 0.49 | | 0.56 | | 1.04 | | | 0.28 |

|  |  | *Offset* | | *Exponent* | |
| --- | --- | --- | --- | --- | --- |
|  |  | *R* | *NR* | *R* | *NR* |
| C5 | *p*-value | 0.40 | **0.01** | 0.99 | **0.003** |
|  | trend |  | **↑** |  | **↑** |
|  | μ_pre_ | 1.48 | 1.76 | 1.86 | 2.11 |
|  | ± σ_pre_ | ±0.42 | ±0.4 | ±0.32 | ±0.64 |
|  | μ_post_ | 1.45 | 1.98 | 1.9 | 2.43 |
|  | ± σ_post_ | ±0.51 | ±0.53 | ±0.34 | ±0.48 |
|  | Cohen’s d | 0.07 | 0.45 | 0.12 | 0.57 |
| C3 | *p*-value | 0.66 | **0.02** | 0.59 | **0.006** |
|  | trend |  | **↑** |  | **↑** |
|  | μ_pre_ | 1.21 | 1.68 | 1.94 | 2.21 |
|  | ± σ_pre_ | ±0.5 | ±0.42 | ±0.34 | ±0.58 |
|  | μ_post_ | 1.17 | 1.88 | 1.99 | 2.5 |
|  | ± σ_post_ | ±0.48 | ±0.6 | ±0.32 | ±0.46 |
|  | Cohen’s d | 0.07 | 0.39 | 0.13 | 0.55 |
| C1 | *p*-value | 0.23 | 0.18 | **0.05** | 0.08 |
|  | trend |  |  | **↑** |  |
|  | μ_pre_ | 1.07 | 1.43 | 2.02 | 2.31 |
|  | ± σ_pre_ | ±0.46 | ±0.42 | ±0.27 | ±0.41 |
|  | μ_post_ | 0.99 | 1.56 | 2.11 | 2.44 |
|  | ± σ_post_ | ±0.42 | ±0.59 | ±0.28 | ±0.47 |
|  | Cohen’s d | 0.18 | 0.26 | 0.31 | 0.23 |
| TP7 | *p*-value | 0.06 | **<0.001** | 0.11 | **<0.001** |
|  | trend |  | **↑** |  | **↑** |
|  | μ_pre_ | 1.61 | 0.94 | 1.89 | 1.86 |
|  | ± σ_pre_ | ±0.39 | ±1.91 | ±0.31 | ±0.90 |
|  | μ_post_ | 1.50 | 2.05 | 1.74 | 2.41 |
|  | ± σ_post_ | ±0.46 | ±0.51 | ±0.38 | ±0.46 |
|  | Cohen’s d | 0.26 | 0.79 | 0.28 | 0.76 |

|  |  | *Offset* | | *Exponent* | |
| --- | --- | --- | --- | --- | --- |
|  |  | *R* | *NR* | *R* | *NR* |
| CP5 | *p*-value | **0.05** | **0.003** | 0.18 | **0.003** |
|  | trend | **↓** | **↑** |  | **↑** |
|  | μ_pre_ | 1.46 | 1.59 | 2.05 | 2.22 |
|  | ± σ_pre_ | ±0.45 | ±0.47 | ±0.24 | ±0.44 |
|  | μ_post_ | 1.30 | 1.83 | 1.99 | 2.44 |
|  | ± σ_post_ | ±0.48 | ±0.50 | ±0.33 | ±0.42 |
|  | Cohen’s d | 0.33 | 0.51 | 0.21 | 0.50 |
| CP3 | *p*-value | 0.51 | **0.07** | 0.11 | 0.45 |
|  | trend |  | **↑** |  |  |
|  | μ_pre_ | 1.09 | 1.46 | 1.97 | 2.16 |
|  | ± σ_pre_ | ±0.49 | ±0.43 | ±0.31 | ±0.6 |
|  | μ_post_ | 1.05 | 1.63 | 2.06 | 2.32 |
|  | ± σ_post_ | ±0.47 | ±0.56 | ±0.28 | ±0.44 |
|  | Cohen’s d | 0.08 | 0.35 | 0.32 | 0.31 |
| CP1 | *p*-value | 0.55 | **0.05** | **0.02** | **0.009** |
|  | trend |  | **↑** | **↑** | **↑** |
|  | μ_pre_ | 0.65 | 1.08 | 1.96 | 2.19 |
|  | ± σ_pre_ | ±0.46 | ±0.41 | ±0.31 | ±0.43 |
|  | μ_post_ | 0.62 | 1.24 | 2.09 | 2.40 |
|  | ± σ_post_ | ±0.43 | ±0.48 | ±0.26 | ±0.44 |
|  | Cohen’s d | 0.09 | 0.38 | 0.46 | 0.48 |
| T5 | *p*-value | **0.006** | **0.02** | **0.005** | 0.07 |
|  | trend | **↓** | **↑** | **↓** |  |
|  | μ_pre_ | 1.62 | 1.72 | 1.99 | 2.17 |
|  | ± σ_pre_ | ±0.41 | ±0.38 | ±0.3 | ±0.40 |
|  | μ_post_ | 1.44 | 1.89 | 1.83 | 2.29 |
|  | ± σ_post_ | ±0.48 | ±0.47 | ±0.40 | ±0.52 |
|  | Cohen’s d | 0.41 | 0.39 | 0.48 | 0.25 |

|  |  | *Offset* | | *Exponent* | |
| --- | --- | --- | --- | --- | --- |
|  |  | *R* | *NR* | *R* | *NR* |
| P5 | *p*-value | 0.12 | 0.09 | 0.44 | **0.02** |
|  | trend |  |  |  | **↑** |
|  | μ_pre_ | 1.43 | 1.60 | 2.08 | 2.14 |
|  | ± σ_pre_ | ±0.45 | ±0.36 | ±0.25 | ±0.53 |
|  | μ_post_ | 1.31 | 1.72 | 2.07 | 2.36 |
|  | ± σ_post_ | ±0.47 | ±0.45 | ±0.34 | ±0.45 |
|  | Cohen’s d | 0.25 | 0.29 | 0.06 | 0.45 |
| P3 | *p*-value | 0.88 | 0.47 | 0.11 | 0.06 |
|  | trend |  |  |  |  |
|  | μ_pre_ | 1.07 | 1.42 | 2.1 | 2.25 |
|  | ± σ_pre_ | ±0.5 | ±0.37 | ±0.24 | ±0.36 |
|  | μ_post_ | 1.08 | 1.49 | 2.19 | 2.38 |
|  | ± σ_post_ | ±0.41 | ±0.44 | ±0.25 | ±0.42 |
|  | Cohen’s d | 0.02 | 0.17 | 0.38 | 0.33 |
| P1 | *p*-value | 0.54 | 0.69 | 0.06 | 0.23 |
|  | trend |  |  |  |  |
|  | μ_pre_ | 0.75 | 1.13 | 2.05 | 2.23 |
|  | ± σ_pre_ | ±0.46 | ±0.39 | ±0.27 | ±0.32 |
|  | μ_post_ | 0.66 | 1.1 | 2.15 | 2.29 |
|  | ± σ_post_ | ±0.60 | ±0.34 | ±0.29 | ±0.37 |
|  | Cohen’s d | 0.17 | 0.09 | 0.37 | 0.17 |
| PO7 | *p*-value | 0.06 | **0.005** | 0.34 | 0.47 |
|  | trend |  | **↑** |  |  |
|  | μ_pre_ | 1.59 | 1.57 | 2.14 | 2.21 |
|  | ± σ_pre_ | ±0.44 | ±0.41 | ±0.24 | ±0.34 |
|  | μ_post_ | 1.45 | 1.79 | 2.09 | 2.14 |
|  | ± σ_post_ | ±0.48 | ±0.48 | ±0.36 | ±0.54 |
|  | Cohen’s d | 0.31 | 0.5 | 0.14 | 0.14 |

|  |  | *Offset* | | *Exponent* | |
| --- | --- | --- | --- | --- | --- |
|  |  | *R* | *NR* | *R* | *NR* |
| PO3 | *p*-value | 0.17 | **<0.001** | 0.80 | 0.06 |
|  | trend |  | **↑** |  |  |
|  | μ_pre_ | 1.25 | 1.22 | 2.16 | 2.13 |
|  | ± σ_pre_ | ±0.557 | ±0.39 | ±0.25 | ±0.43 |
|  | μ_post_ | 1.13 | 1.49 | 2.22 | 2.28 |
|  | ± σ_post_ | ±0.53 | ±0.39 | ±0.29 | ±0.45 |
|  | Cohen’s d | 0.21 | 0.7 | 0.23 | 0.33 |
| O1 | *p*-value | 0.16 | **0.01** | 0.87 | 0.78 |
|  | trend |  | **↑** |  |  |
|  | μ_pre_ | 1.43 | 1.53 | 2.15 | 2.18 |
|  | ± σ_pre_ | ±0.50 | ±0.34 | ±0.27 | ±0.41 |
|  | μ_post_ | 1.34 | 1.68 | 2.19 | 2.18 |
|  | ± σ_post_ | ±0.41 | ±0.44 | ±0.28 | ±0.55 |
|  | Cohen’s d | 0.2 | 0.41 | 0.14 | 0.01 |
| FPZ | *p*-value | 0.08 | **<0.001** | 0.39 | **<0.001** |
|  | trend |  | **↑** |  | **↑** |
|  | μ_pre_ | 1.86 | 2.06 | 2.13 | 2.32 |
|  | ± σ_pre_ | ±0.49 | ±0.56 | ±0.27 | ±0.49 |
|  | μ_post_ | 1.741 | 2.46 | 2.08 | 2.61 |
|  | ± σ_post_ | ±0.41 | ±0.6 | ±0.32 | ±0.48 |
|  | Cohen’s d | 0.27 | 0.69 | 0.17 | 0.6 |
| AFZ | *p*-value | **0.03** | **<0.001** | 0.85 | **<0.001** |
|  | trend | **↓** | **↑** |  | **↑** |
|  | μ_pre_ | 1.81 | 2.08 | 2.16 | 2.31 |
|  | ± σ_pre_ | ±0.52 | ±0.43 | ±0.27 | ±0.48 |
|  | μ_post_ | 1.66 | 2.37 | 2.14 | 2.61 |
|  | ± σ_post_ | ±0.41 | ±0.63 | ±0.30 | ±0.46 |
|  | Cohen’s d | 0.34 | 0.55 | 0.06 | 0.64 |

|  |  | *Offset* | | *Exponent* | |
| --- | --- | --- | --- | --- | --- |
|  |  | *R* | *NR* | *R* | *NR* |
| FZ | *p*-value | 0.09 | **0.003** | 0.69 | **<0.001** |
|  | trend |  | **↑** |  | **↑** |
|  | μ_pre_ | 1.62 | 1.93 | 2.15 | 2.285 |
|  | ± σ_pre_ | ±0.50 | ±0.45 | ±0.25 | ±0.44 |
|  | μ_post_ | 1.48 | 2.22 | 2.12 | 2.56 |
|  | ± σ_post_ | ±0.45 | ±0.65 | ±0.31 | ±0.46 |
|  | Cohen’s d | 0.3 | 0.52 | 0.10 | 0.63 |
| FCZ | *p*-value | 0.45 | 0.29 | 0.72 | **0.05** |
|  | trend |  |  |  | **↑** |
|  | μ_pre_ | 1.43 | 1.85 | 2.1 | 2.28 |
|  | ± σ_pre_ | ±0.49 | ±0.48 | ±0.23 | ±0.40 |
|  | μ_post_ | 1.39 | 1.92 | 2.12 | 2.43 |
|  | ± σ_post_ | ±0.47 | ±0.61 | ±0.31 | ±0.47 |
|  | Cohen’s d | 0.1 | 0.14 | 0.07 | 0.35 |
| CZ | *p*-value | 0.44 | 0.62 | 0.12 | **0.05** |
|  | trend |  |  |  | **↑** |
|  | μ_pre_ | 0.99 | 1.43 | 2.05 | 2.1 |
|  | ± σ_pre_ | ±0.46 | ±0.40 | ±0.22 | ±0.49 |
|  | μ_post_ | 0.94 | 1.48 | 2.13 | 2.39 |
|  | ± σ_post_ | ±0.45 | ±0.62 | ±0.3 | ±0.50 |
|  | Cohen’s d | 0.10 | 0.11 | 0.30 | 0.39 |
| CPZ | *p*-value | 0.10 | 0.63 | 0.69 | **<0.001** |
|  | trend |  |  |  | **↑** |
|  | μ_pre_ | -0.46 | -0.37 | 1.77 | 1.62 |
|  | ± σ_pre_ | ±1.08 | ±1.53 | ±0.39 | ±0.61 |
|  | μ_post_ | -0.72 | 0.04 | 1.75 | 2.10 |
|  | ± σ_post_ | ±0.84 | ±0.81 | ±0.32 | ±0.45 |
|  | Cohen’s d | 0.27 | 0.34 | 0.07 | 0.90 |

|  |  | *Offset* | | *Exponent* | |
| --- | --- | --- | --- | --- | --- |
|  |  | *R* | *NR* | *R* | *NR* |
| PZ | *p*-value | **0.01** | **<0.001** | 0.46 | **<0.001** |
|  | trend | **↓** | **↑** |  | **↑** |
|  | μ_pre_ | -0.24 | -0.231 | 1.81 | 1.77 |
|  | ± σ_pre_ | ±0.94 | ±1.47 | ±0.4 | ±0.35 |
|  | μ_post_ | -0.68 | 0.7 | 1.77 | 2.25 |
|  | ± σ_post_ | ±1.06 | ±0.45 | ±0.43 | ±0.41 |
|  | Cohen’s d | 0.45 | 0.852 | 0.12 | 1.26 |
| POZ | *p*-value | 0.23 | 0.71 | 0.94 | 0.27 |
|  | trend |  |  |  |  |
|  | μ_pre_ | 1.10 | 1.52 | 2.05 | 2.26 |
|  | ± σ_pre_ | ±0.48 | ±0.40 | ±0.26 | ±0.36 |
|  | μ_post_ | 1.02 | 1.5 | 2.08 | 2.34 |
|  | ± σ_post_ | ±0.48 | ±0.42 | ±0.34 | ±0.48 |
|  | Cohen’s d | 0.17 | 0.05 | 0.1 | 0.2 |
| OZ | *p*-value | **0.01** | 0.07 | 0.46 | 0.30 |
|  | trend | **↓** |  |  |  |
|  | μ_pre_ | 1.4 | 1.66 | 2.12 | 2.25 |
|  | ± σ_pre_ | ±0.42 | ±0.39 | ±0.24 | ±0.39 |
|  | μ_post_ | 1.21 | 1.80 | 2.09 | 2.32 |
|  | ± σ_post_ | ±0.43 | ±0.44 | ±0.33 | ±0.51 |
|  | Cohen’s d | 0.44 | 0.35 | 0.10 | 0.15 |
| FP2 | *p*-value | 0.07 | **<0.001** | 0.18 | **<0.001** |
|  | trend |  | **↑** |  | **↑** |
|  | μ_pre_ | 1.92 | 2.16 | 2.08 | 2.30 |
|  | ± σ_pre_ | ±0.52 | ±0.40 | ±0.32 | ±0.42 |
|  | μ_post_ | 1.77 | 2.44 | 2.01 | 2.59 |
|  | ± σ_post_ | ±0.44 | ±0.59 | ±0.34 | ±0.46 |
|  | Cohen’s d | 0.32 | 0.56 | 0.22 | 0.65 |

|  |  | *Offset* | | *Exponent* | |
| --- | --- | --- | --- | --- | --- |
|  |  | *R* | *NR* | *R* | *NR* |
| AF8 | *p*-value | **<0.001** | **0.003** | **<0.001** | **<0.001** |
|  | trend | **↓** | **↑** | **↓** | **↑** |
|  | μ_pre_ | 2.01 | 2.17 | 2.06 | 2.2 |
|  | ± σ_pre_ | ±0.47 | ±0.40 | ±0.33 | ±0.47 |
|  | μ_post_ | 1.79 | 2.36 | 1.92 | 2.45 |
|  | ± σ_post_ | ±0.42 | ±0.58 | ±0.34 | ±0.50 |
|  | Cohen’s d | 0.51 | 0.40 | 0.44 | 0.52 |
| AF4 | *p*-value | 0.16 | **<0.001** | 0.99 | **<0.001** |
|  | trend |  | **↑** |  | **↑** |
|  | μ_pre_ | 1.81 | 2.09 | 2.1 | 2.30 |
|  | ± σ_pre_ | ±0.54 | ±0.45 | ±0.31 | ±0.49 |
|  | μ_post_ | 1.69 | 2.39 | 2.11 | 2.59 |
|  | ± σ_post_ | ±0.41 | ±0.6 | ±0.28 | ±0.46 |
|  | Cohen’s d | 0.26 | 0.58 | 0.02 | 0.60 |
| F8 | *p*-value | 0.06 | **0.005** | 0.12 | 0.08 |
|  | trend |  | **↑** |  |  |
|  | μ_pre_ | 1.95 | 2.07 | 1.91 | 2.19 |
|  | ± σ_pre_ | ±0.54 | ±0.42 | ±0.53 | ±0.46 |
|  | μ_post_ | 1.77 | 2.25 | 1.81 | 2.3 |
|  | ± σ_post_ | ±0.39 | ±0.48 | ±0.38 | ±0.45 |
|  | Cohen’s d | 0.4 | 0.41 | 0.21 | 0.23 |
| F6 | *p*-value | **<0.001** | **0.05** | **0.005** | 0.07 |
|  | trend | **↓** | **↑** | **↓** |  |
|  | μ_pre_ | 1.85 | 2.03 | 1.99 | 2.27 |
|  | ± σ_pre_ | ±0.5 | ±0.43 | ±0.4 | ±0.45 |
|  | μ_post_ | 1.55 | 2.18 | 1.79 | 2.41 |
|  | ± σ_post_ | ±0.43 | ±0.51 | ±0.38 | ±0.50 |
|  | Cohen’s d | 0.65 | 0.30 | 0.50 | 0.29 |

|  |  | *Offset* | | *Exponent* | |
| --- | --- | --- | --- | --- | --- |
|  |  | *R* | *NR* | *R* | *NR* |
| F4 | *p*-value | **0.04** | **0.02** | **0.05** | **0.01** |
|  | trend | **↓** | **↑** | **↓** | **↑** |
|  | μ_pre_ | 1.66 | 1.98 | 2.02 | 2.28 |
|  | ± σ_pre_ | ±0.55 | ±0.44 | ±0.41 | ±0.48 |
|  | μ_post_ | 1.48 | 2.14 | 1.93 | 2.48 |
|  | ± σ_post_ | ±0.42 | ±0.62 | ±0.27 | ±0.48 |
|  | Cohen’s d | 0.36 | 0.30 | 0.25 | 0.41 |
| F2 | *p*-value | 0.09 | 0.11 | 0.28 | **0.02** |
|  | trend |  |  |  | **↑** |
|  | μ_pre_ | 1.61 | 2.03 | 2.07 | 2.33 |
|  | ± σ_pre_ | ±0.54 | ±0.49 | ±0.36 | ±0.45 |
|  | μ_post_ | 1.46 | 2.13 | 2.03 | 2.51 |
|  | ± σ_post_ | ±0.44 | ±0.62 | ±0.27 | ±0.47 |
|  | Cohen’s d | 0.30 | 0.17 | 0.15 | 0.40 |
| FT8 | *p*-value | **0.01** | **<0.001** | **0.005** | **0.009** |
|  | trend | **↓** | **↑** | **↓** | **↑** |
|  | μ_pre_ | 1.89 | 1.88 | 1.91 | 2.10 |
|  | ± σ_pre_ | ±0.56 | ±0.44 | ±0.56 | ±0.49 |
|  | μ_post_ | 1.63 | 2.23 | 1.67 | 2.31 |
|  | ± σ_post_ | ±0.45 | ±0.46 | ±0.47 | ±0.47 |
|  | Cohen’s d | 0.50 | 0.78 | 0.46 | 0.43 |
| FC6 | *p*-value | **<0.001** | **0.01** | **<0.001** | 0.08 |
|  | trend | **↓** | **↑** | **↓** |  |
|  | μ_pre_ | 1.7 | 1.82 | 1.94 | 2.17 |
|  | ± σ_pre_ | ±0.56 | ±0.46 | ±0.48 | ±0.47 |
|  | μ_post_ | 1.40 | 2.02 | 1.65 | 2.32 |
|  | ± σ_post_ | ±0.43 | ±0.51 | ±0.40 | ±0.52 |
|  | Cohen’s d | 0.59 | 0.41 | 0.64 | 0.3 |

|  |  | *Offset* | | *Exponent* | |
| --- | --- | --- | --- | --- | --- |
|  |  | *R* | *NR* | *R* | *NR* |
| FC4 | *p*-value | **0.02** | 0.12 | **<0.001** | 0.12 |
|  | trend | **↓** |  | **↓** |  |
|  | μ_pre_ | 1.49 | 1.75 | 2.02 | 2.25 |
|  | ± σ_pre_ | ±0.54 | ±0.4 | ±0.41 | ±0.44 |
|  | μ_post_ | 1.27 | 1.89 | 1.84 | 2.38 |
|  | ± σ_post_ | ±0.44 | ±0.47 | ±0.32 | ±0.48 |
|  | Cohen’s d | 0.44 | 0.31 | 0.50 | 0.27 |
| FC2 | *p*-value | 0.07 | 0.50 | 0.17 | 0.06 |
|  | trend |  |  |  |  |
|  | μ_pre_ | 1.42 | 1.80 | 2.05 | 2.20 |
|  | ± σ_pre_ | ±0.53 | ±0.44 | ±0.375 | ±0.56 |
|  | μ_post_ | 1.27 | 1.86 | 1.99 | 2.42 |
|  | ± σ_post_ | ±0.44 | ±0.58 | ±0.27 | ±0.47 |
|  | Cohen’s d | 0.31 | 0.12 | 0.16 | 0.43 |
| T4 | *p*-value | **0.006** | **<0.001** | **<0.001** | **0.003** |
|  | trend | **↓** | **↑** | **↓** | **↑** |
|  | μ_pre_ | 1.75 | 1.83 | 1.83 | 1.99 |
|  | ± σ_pre_ | ±0.44 | ±0.42 | ±0.35 | ±0.53 |
|  | μ_post_ | 1.52 | 2.14 | 1.52 | 2.28 |
|  | ± σ_post_ | ±0.45 | ±0.57 | ±0.50 | ±0.62 |
|  | Cohen’s d | 0.52 | 0.62 | 0.73 | 0.49 |
| C6 | *p*-value | **<0.001** | **<0.001** | **<0.001** | **0.02** |
|  | trend | **↓** | **↑** | **↓** | **↑** |
|  | μ_pre_ | 1.57 | 1.65 | 1.98 | 2.18 |
|  | ± σ_pre_ | ±0.48 | ±0.50 | ±0.40 | ±0.51 |
|  | μ_post_ | 1.31 | 1.96 | 1.66 | 2.39 |
|  | ± σ_post_ | ±0.50 | ±0.51 | ±0.46 | ±0.51 |
|  | Cohen’s d | 0.52 | 0.63 | 0.75 | 0.41 |

|  |  | *Offset* | | *Exponent* | |
| --- | --- | --- | --- | --- | --- |
|  |  | *R* | *NR* | *R* | *NR* |
| C4 | *p*-value | 0.09 | 0.04 | **0.02** | 0.07 |
|  | trend |  |  | **↓** |  |
|  | μ_pre_ | 1.27 | 1.64 | 1.99 | 2.29 |
|  | ± σ_pre_ | ±0.51 | ±0.34 | ±0.37 | ±0.41 |
|  | μ_post_ | 1.14 | 1.78 | 1.9 | 2.42 |
|  | ± σ_post_ | ±0.47 | ±0.38 | ±0.32 | ±0.43 |
|  | Cohen’s d | 0.27 | 0.38 | 0.3 | 0.32 |
| C2 | *p*-value | 0.50 | 0.18 | 0.99 | 0.12 |
|  | trend |  |  |  |  |
|  | μ_pre_ | 1.07 | 1.57 | 2.02 | 2.258 |
|  | ± σ_pre_ | ±0.49 | ±0.37 | ±0.36 | ±0.44 |
|  | μ_post_ | 1.02 | 1.52 | 2.05 | 2.4 |
|  | ± σ_post_ | ±0.44 | ±0.49 | ±0.28 | ±0.45 |
|  | Cohen’s d | 0.10 | 0.12 | 0.09 | 0.33 |
| TP8 | *p*-value | **0.006** | **0.010** | **<0.001** | **0.003** |
|  | trend | **↓** | **↑** | **↓** | **↑** |
|  | μ_pre_ | 1.67 | 1.91 | 1.96 | 2.16 |
|  | ± σ_pre_ | ±0.39 | ±0.35 | ±0.28 | ±0.42 |
|  | μ_post_ | 1.47 | 2.16 | 1.75 | 2.43 |
|  | ± σ_post_ | ±0.43 | ±0.60 | ±0.47 | ±0.58 |
|  | Cohen’s d | 0.49 | 0.51 | 0.54 | 0.52 |
| CP6 | *p*-value | **0.02** | **0.005** | **<0.001** | **0.009** |
|  | trend | **↓** | **↑** | **↓** | **↑** |
|  | μ_pre_ | 1.42 | 1.63 | 2.08 | 2.21 |
|  | ± σ_pre_ | ±0.5 | ±0.43 | ±0.33 | ±0.46 |
|  | μ_post_ | 1.23 | 1.89 | 1.88 | 2.44 |
|  | ± σ_post_ | ±0.49 | ±0.58 | ±0.40 | ±0.54 |
|  | Cohen’s d | 0.4 | 0.52 | 0.55 | 0.46 |

|  |  | *Offset* | | *Exponent* | |
| --- | --- | --- | --- | --- | --- |
|  |  | *R* | *NR* | *R* | *NR* |
| CP4 | *p*-value | 0.12 | **0.007** | 0.39 | **0.03** |
|  | trend |  | **↑** |  | **↑** |
|  | μ_pre_ | 1.1 | 0.69 | 2.03 | 1.99 |
|  | ± σ_pre_ | ±0.54 | ±1.78 | ±0.36 | ±0.93 |
|  | μ_post_ | 0.97 | 1.6 | 1.99 | 2.43 |
|  | ± σ_post_ | ±0.49 | ±0.49 | ±0.32 | ±0.48 |
|  | Cohen’s d | 0.24 | 0.70 | 0.12 | 0.6 |
| CP2 | *p*-value | 0.40 | **0.010** | 0.52 | 0.47 |
|  | trend |  | **↓** |  |  |
|  | μ_pre_ | 0.71 | 1.2 | 2.03 | 2.26 |
|  | ± σ_pre_ | ±0.45 | ±0.33 | ±0.34 | ±0.38 |
|  | μ_post_ | 0.65 | 1.06 | 2.09 | 2.31 |
|  | ± σ_post_ | ±0.39 | ±0.39 | ±0.25 | ±0.41 |
|  | Cohen’s d | 0.15 | 0.37 | 0.21 | 0.13 |
| T6 | *p*-value | **0.02** | 0.36 | 0.32 | 0.23 |
|  | trend | **↓** |  |  |  |
|  | μ_pre_ | 1.49 | 1.9 | 1.95 | 2.25 |
|  | ± σ_pre_ | ±0.4 | ±0.34 | ±0.27 | ±0.49 |
|  | μ_post_ | 1.34 | 2.04 | 1.88 | 2.36 |
|  | ± σ_post_ | ±0.44 | ±0.63 | ±0.41 | ±0.61 |
|  | Cohen’s d | 0.38 | 0.29 | 0.2 | 0.21 |
| P6 | *p*-value | 0.19 | **0.02** | 0.29 | **0.02** |
|  | trend |  | **↑** |  | **↑** |
|  | μ_pre_ | 1.29 | 1.67 | 2.05 | 2.24 |
|  | ± σ_pre_ | ±0.46 | ±0.39 | ±0.29 | ±0.46 |
|  | μ_post_ | 1.20 | 1.91 | 1.97 | 2.44 |
|  | ± σ_post_ | ±0.47 | ±0.6 | ±0.46 | ±0.54 |
|  | Cohen’s d | 0.18 | 0.47 | 0.20 | 0.4 |

|  |  | *Offset* | | *Exponent* | |
| --- | --- | --- | --- | --- | --- |
|  |  | *R* | *NR* | *R* | *NR* |
| P4 | *p*-value | 0.08 | **0.02** | 0.13 | 0.23 |
|  | trend |  | **↑** |  |  |
|  | μ_pre_ | 1.07 | 1.32 | 2.105 | 2.281 |
|  | ± σ_pre_ | ±0.45 | ±0.52 | ±0.345 | ±0.455 |
|  | μ_post_ | 0.91 | 1.56 | 2.029 | 2.410 |
|  | ± σ_post_ | ±0.47 | ±0.59 | ±0.349 | ±0.532 |
|  | Cohen’s d | 0.35 | 0.44 | 0.219 | 0.262 |
| P2 | *p*-value | 0.86 | **0.005** | 0.26 | 0.06 |
|  | trend |  | **↑** |  |  |
|  | μ_pre_ | 0.73 | 1.09 | 2.008 | 2.241 |
|  | ± σ_pre_ | ±0.5 | ±0.45 | ±0.358 | ±0.415 |
|  | μ_post_ | 0.75 | 1.31 | 2.110 | 2.403 |
|  | ± σ_post_ | ±0.49 | ±0.48 | ±0.245 | ±0.487 |
|  | Cohen’s d | 0.04 | 0.5 | 0.333 | 0.358 |
| PO8 | *p*-value | **0.05** | **0.07** | 0.15 | 0.27 |
|  | trend | **↓** | **↑** |  |  |
|  | μ_pre_ | 1.44 | 1.81 | 2.034 | 2.237 |
|  | ± σ_pre_ | ±0.41 | ±0.42 | ±0.241 | ±0.562 |
|  | μ_post_ | 1.3 | 2.02 | 1.931 | 2.371 |
|  | ± σ_post_ | ±0.47 | ±0.60 | ±0.461 | ±0.601 |
|  | Cohen’s d | 0.31 | 0.41 | 0.280 | 0.230 |
